# The effects of ankle stiffness on mechanics and energetics of walking with added loads: a prosthetic emulator study

**DOI:** 10.1186/s12984-019-0621-x

**Published:** 2019-11-21

**Authors:** Erica A. Hedrick, Philippe Malcolm, Jason M. Wilken, Kota Z. Takahashi

**Affiliations:** 1 Department of Biomechanics, University of Nebraska at Omaha, Omaha, NE USA; 2 Department of Physical Therapy & Rehabilitation Science, University of Iowa, Iowa City, Iowa, USA

**Keywords:** Biomechanics, Load carriage, Prosthetics, Quasi-stiffness, Foot, Locomotion

## Abstract

**Background:**

The human ankle joint has an influential role in the regulation of the mechanics and energetics of gait. The human ankle can modulate its joint ‘quasi-stiffness’ (ratio of plantarflexion moment to dorsiflexion displacement) in response to various locomotor tasks (e.g., load carriage). However, the direct effect of ankle stiffness on metabolic energy cost during various tasks is not fully understood. The purpose of this study was to determine how net metabolic energy cost was affected by ankle stiffness while walking under different force demands (i.e., with and without additional load).

**Methods:**

Individuals simulated an amputation by using an immobilizer boot with a robotic ankle-foot prosthesis emulator. The prosthetic emulator was controlled to follow five ankle stiffness conditions, based on literature values of human ankle quasi-stiffness. Individuals walked with these five ankle stiffness settings, with and without carrying additional load of approximately 30% of body mass (i.e., ten total trials).

**Results:**

Within the range of stiffness we tested, the highest stiffness minimized metabolic cost for both load conditions, including a ~ 3% decrease in metabolic cost for an increase in stiffness of about 0.0480 Nm/deg/kg during normal (no load) walking. Furthermore, the highest stiffness produced the least amount of prosthetic ankle-foot positive work, with a difference of ~ 0.04 J/kg from the highest to lowest stiffness condition. Ipsilateral hip positive work did not significantly change across the no load condition but was minimized at the highest stiffness for the additional load conditions. For the additional load conditions, the hip work followed a similar trend as the metabolic cost, suggesting that reducing positive hip work can lower metabolic cost.

**Conclusion:**

While ankle stiffness affected the metabolic cost for both load conditions, we found no significant interaction effect between stiffness and load. This may suggest that the importance of the human ankle’s ability to change stiffness during different load carrying tasks may not be driven to minimize metabolic cost. A prosthetic design that can modulate ankle stiffness when transitioning from one locomotor task to another could be valuable, but its importance likely involves factors beyond optimizing metabolic cost.

## Background

The human ankle joint has an important influence on mechanics and energetics of gait. Specifically, the role of the muscles acting at the ankle joint during normal walking is to provide body support, aid in forward propulsion, and to initiate leg swing [1]. The peak ankle joint power can be ~ 2.5 W/kg, which is greater than the maximum power produced by the knee joint and hip joint [2, 3]. Individuals with impaired ankle function (e.g., older adults, individuals who have survived a stroke) or individuals with artificial ankles (e.g., individuals with an amputation) have to compensate for the diminished ankle joint power with proximal muscles such as the hip joint [4–8]. Thus, preserving natural ankle joint functions is important for rehabilitation and/or assistive devices since compensations via proximal muscles can often lead to an increase in metabolic cost of walking [9–11].

One important feature of the human ankle joint is the regulation of ‘quasi-stiffness,’ which hereby will be referred to as stiffness. The human ankle stiffness is defined as the slope of the moment-angle relationship of the joint, or the ratio of the ankle moment to angular displacement [12–14]. This stiffness can be quantified in different phases during the gait cycle [13], including when the ankle joint is dorsiflexing while applying a plantarflexion moment (i.e., dorsiflexion stiffness). There appears to be an optimal level of ankle dorsiflexion stiffness to aid the shank as it rocks over the foot, which has been supported through several studies involving prosthetic ankles. If the prosthetic ankle joint is too compliant, then the joint may not provide enough plantarflexion moment to adequately support the body upright [6, 15, 16]. On the other hand, if the prosthetic ankle joint is too stiff, there would be excessive resistance to dorsiflexion motion, which would prohibit the shank’s progression [6].

Numerous studies have shown that humans can alter ankle dorsiflexion stiffness in response to changes in mechanical demands of walking [17–19], most likely through modulation of muscle activation. As walking speed increases or when walking uphill, the human ankle joint stiffness increases due to the plantarflexion moment increasing and the dorsiflexion angle decreasing [17, 18]. Additionally, the human ankle joint stiffness increases when individuals walk with additional load [18, 20]. Kern et al. found that the human ankle stiffness, normalized to body mass, increased by about 13% when walking with 30% additional body mass [20]. All of these studies show the human’s capacity to modulate ankle joint stiffness in response to the mechanical demand of the task. However, the functional importance of such ability to modulate stiffness is unclear. Due to the human ankle’s purported role in minimizing metabolic energy expenditure during locomotion [21], it is possible that modulating stiffness when transitioning from one locomotion task to another (e.g., normal walking to load carrying) could preserve energy expenditure across the various locomotor demands. Yet, there are currently no studies that have directly linked the ankle’s ability to modulate stiffness and their role in minimizing metabolic energy expenditure across various locomotor tasks. Such knowledge would contribute to the overall structure-function relationship of the human ankle and could also inform designs of wearable devices (e.g., prostheses) intended to emulate biological function.

Studies involving lower-limb ankle-foot prostheses have provided valuable insights on the role of stiffness in regulating metabolic energy during walking. There have been many studies done to determine what the best prosthetic ankle or foot stiffness is for lowering metabolic cost and improving gait for individuals with amputation [6, 15, 16, 22, 23]. Major et al. showed that a lower dorsiflexion ankle stiffness (relative to commercially-available prostheses) reduces the vertical ground reaction force during the loading phase of the prosthetic stance as well as the net metabolic cost [15]. Fey et al. showed that lower foot stiffness can also increase the amount of energy stored and returned, contributing to greater forward propulsion and assisting swing initiation [6]. Zelik et al. had individuals walk with three different spring stiffnesses in prosthetic feet and found that an intermediate spring stiffness had the lowest metabolic energy [24], suggesting a quadratic relationship between prosthetic stiffness and metabolic cost. While these studies indicate that the stiffness of the ankle joint or prosthetic foot plays a role in regulating metabolic energy during normal walking, the role that this stiffness has in regulating metabolic energy across different walking conditions and demands (e.g., walking with added loads) is unknown. When individuals with an amputation walk with additional loads, they have altered gait mechanics [25] and expend more metabolic energy than healthy controls [26]. Thus, determining whether a prosthesis should be able to change stiffness across different walking conditions may be important, which could warrant recent developments in micro-processor-controlled prostheses that can modulate stiffness [27, 28].

**Fig. 1:**
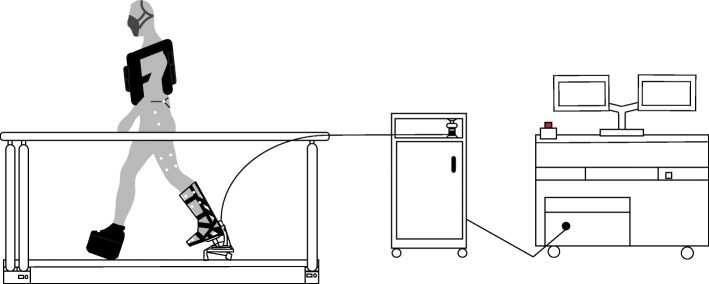
Experimental setup. The prosthesis emulator is tethered to an off-board motor and controlled via a computer interfaced with MATLAB and Simulink. The desired stiffness can be entered into the software, which allows the ankle stiffness to be systematically varied during the dorsiflexion phase and push-off. The protocol tested five different prosthetic ankle stiffness (based on literature-values of human ankle stiffness) with and without a weighted vest (~ 30% of body mass). The participants wore a lift shoe on the contralateral leg

The purpose of this study was to determine how net metabolic energy cost was affected by ankle stiffness while walking with different mechanical demands (i.e., with and without additional load). Walking with an additional load directly increases metabolic cost [29]. As a proof of concept, this study involved individuals with a simulated amputation by using an immobilizer boot with the prosthesis, which has been used in various other studies [24, 30–33] (Fig. 1). We used a robotic prosthetic emulator, which simulated an elastic prosthesis with a range of ankle joint stiffnesses around a typical human ankle stiffness value during walking with and without additional loads [14, 18, 20, 34]. We hypothesized that the lowest stiffness would minimize metabolic cost for walking without added load. We also hypothesized that the stiffness that minimized metabolic cost during the load carriage would be greater compared to the no load conditions, since the human ankle increases its stiffness when walking with added load [18, 20]. Furthermore, we hypothesized that the lowest stiffness would maximize prosthetic positive ankle-foot work and minimize ipsilateral hip positive work. Lastly, we hypothesized that maximizing prosthetic ankle-foot work and minimizing ipsilateral positive hip work would require a greater prosthesis stiffness during the load carrying conditions than in the no load conditions. The findings of this research could help uncover the importance of the human ankle’s ability to modulate joint stiffness across locomotor tasks, and could also inform how prostheses should change ankle stiffness based on walking demands.

## Methods

### Robotic prosthetic ankle emulator

Participants simulated an amputation by wearing the prosthesis with an immobilizer boot attached. An experimental ankle-foot prosthesis emulator (HuMoTech, Pittsburgh, PA) was used to systematically vary resistance to ankle dorsiflexion (i.e., stiffness) (Fig. 1). A similar device has been used in multiple previous studies [31, 33, 35]. All mechanical features of the prosthesis, including size, mass, heel stiffness, and alignment, remained unchanged across conditions. The mass of the prosthesis, simulator boot, and lift shoe was 0.96 kg, 1.6 kg, and 1.1 kg, respectively. The length of the prosthetic foot was 0.24 m, the heel of the prosthesis was 0.070 m behind the ankle joint, and the total added leg length while walking on the prosthesis and simulator boot was 0.13 m. The prosthesis simulated a passive prosthesis that provided net work near zero or slightly net negative. The prosthesis was tethered to an off-board motor and computer, and the tether was supported near the participant to minimize its interference when participants were walking. Participants wore the ankle-foot prosthesis with the simulator boot on their right leg. A lift shoe (length 0.29 m or 0.31 m) with a rocker bottom was worn on the left foot to keep leg lengths equal [31].

To control the prosthetic ankle joint stiffness, adjustments were made electronically using MATLAB/Simulink software (MathWorks, Natick, MA). In order to create the desired moment-angle relationship, we entered two moment and angle value-pairs into the software to define a linear slope (i.e., stiffness) (Additional file 1: Figure S1). For the first pair, we always entered a desired plantarflexion moment of 0 Nm at 0 degrees dorsiflexion. For the second pair we entered a condition-specific non-zero dorsiflexion value and plantarflexion moment value. The control software would then apply torques as a function of dorsiflexion angle based on a linear fit through these two points, depending on the prosthesis angle. When the dorsiflexion angle would be greater than the dorsiflexion from the second value-pair, the prosthesis would simply apply higher moments from the extrapolated fit between the two value-pairs. The hardware and off-board motor tried to match the desired moment-angle relationship created in the software. The ankle dorsiflexion stiffness was quantified similar to the calculation seen in previous studies, in which they used the slope of the best fit line of the moment-angle curve [12, 20].

### Participants

Fourteen healthy young adults (individuals without transtibial amputation) (1 female, 13 males; ages 25.71 ± 3.06 yrs.; height 1.75 ± 0.05 m; body mass 75.07 ± 6.22 kg; mean ± sd.) volunteered to participate in the study. Healthy was defined as: free of musculoskeletal or pathological problems including cardiovascular and neurological disorders. Participants did not have any past injuries or surgeries that affected their gait; any current pain in the neck, back, or shoulders; or any current medication that may affect temporal spatial awareness, joint or muscle stiffness and cognitive function. They were able to carry 30% of their body mass as added weight. Since the weight limit of the prosthesis was 113.4 kg, all individuals were under 87.23 kg and had a body mass index under 30 kg/m^2^. These conditions were screened using a medical-history form. The study was conducted at the University of Nebraska at Omaha (UNO) under the approval of the Institutional Review Board of the University of Nebraska Medical Center. Each participant provided written consent before being screened for inclusion and exclusion criteria.

### Experimental design

#### Overview

This experiment consisted of 10 conditions each visit. The 10 conditions included five different prosthetic stiffness settings and two different load carrying conditions. All 10 conditions were repeated on three different days to account for any learning effects. There were 24 h to 72 h in between each session. This study set up was done in a previous study using this device [31]. Reported data are from the final visit.

The five stiffness settings were 0.0928, 0.1044 0.1160, 0.1276 and 0.1392 Nm/deg/kg. Since the goal of this study was to understand the functional importance of human ankle’s ability to modulate stiffness, we selected stiffness values near the typical human ankle during normal walking, as well as during load carriage. Literature values for typical human ankle stiffness (for no load walking) have ranged from ~ 0.089 to ~ 0.1077 Nm/deg/kg [14, 20, 34]. During load carriage, the human ankle stiffness can range from ~ 0.093 Nm/deg/kg while carrying 15% of body mass, ~ 0.100 Nm/deg/kg while carrying 30% of body mass, and ~ 0.127 Nm/deg/kg while carrying 61% of load [18, 20]. Thus, our five stiffness settings are within the range of typical human ankle stiffness values during walking with and without carrying additional loads [14, 18, 20, 34].

The load carrying conditions were an additional 0% (no additional load) and 30% of the participant’s body mass. 30% additional body mass was chosen because previous studies have shown that metabolic cost increases with added body mass in an almost linear relationship [29, 36, 37]. Therefore, 30% added body mass would be enough to see a noticeable difference in metabolic cost between the two conditions. The prosthesis used had a weight limit, so we wanted to stay within the limits of the prosthesis, while using the highest possible load. Additionally, a previous study from our lab examined how human ankle modulates stiffness when walking with up to 30% additional body mass [20]*,* which provided further justifications for the stiffness levels used for this current study*.* The 30% additional body mass was symmetrically distributed around the participant’s core, in a weighted vest, with 2.5 kg weights. Since the weight was in incremental amounts, the participants wore the weight that was closest to 30% of their body weight. However, the actual weight percentage ranged from 29 to 34% of the participant’s body mass. Conditions were blocked randomized, in which the no additional load conditions were first, followed by the additional load conditions. The five stiffness conditions were randomized within each load carrying condition.

#### Protocol

Participants were asked to fast 3 h before the data collection, as to not affect the metabolic cost estimates. The visit started with obtaining consent and then taking anthropometric measurements using a caliper, including lower limb segment lengths and width. Prior to data collection, participants were asked to put on a tight-fitting spandex suit to limit movement noise from the reflective markers. A six-degree-of-freedom marker set was used to track the motion of the lower extremities and the prosthesis [38]. This marker set had been shown to reliably assess gait performance over multiple sessions [38]. Five additional markers were placed on the prosthesis emulator, with three on the body of the prosthesis and two on the ankle joint (lateral and medial). Three-dimensional lower limb kinematic data were captured using an eight-camera motion capture system (VICON, Oxford, UK), as participants walked on an instrumented treadmill (Bertec, Columbus, OH) to capture limb kinetics.

Before the first trial, a 7-min recording of the metabolic rate was taken as the participant stood quietly on the instrumented treadmill. For each trial, the participant walked for 6 min at a speed of 1.25 m/s. The treadmill started at 0.8 m/s and gradually increased speed up to 1.25 m/s. Once at 1.25 m/s, the 6 min started. During all conditions, the maximum torque and ankle range of motion of the prosthesis were monitored to make sure either were not reaching the hardware limit. Breath-by-breath gas exchange measurements were recorded for indirect calorimetry calculations of metabolic cost (True One, Parvo Medics). This metabolic system has been shown to provide reliable results for gas exchange measurements [39]. Data from the last 2 min were used for the indirect calorimetry calculations in order to use steady state data. Oxygen and carbon dioxide volume over time was plotted and visually checked to confirm steady state had been reached. Kinematic and kinetic data were recorded during the last minute of the trial. Immediately after the trial ended, the participants were asked about their perception of the condition on a scale of − 10 to 10, with 0 corresponding to the same as their normal walking, − 10 corresponding to cannot walk, and + 10 corresponding to walking is effortless and significantly easier than normal. This questionnaire regarding perception was done in a similar previous study [33]. A rest period of at least 5 min occurred between each condition to allow for recovery and to minimize fatigue.

### Data analysis

#### Metabolic cost

Standard calculations derived by Brockway [40] were used to calculate whole-body metabolic power. Energy expenditure was estimated by using the volume of oxygen consumption and carbon dioxide production. Net metabolic power was defined as the metabolic power during walking minus the metabolic power during a quiet standing trial. Net metabolic power was normalized by dividing the power by the participants’ biological body mass.

#### Kinematic and kinetic data

Kinematic and kinetic data were sampled at 250 and 1000 Hz, respectively, and filtered with a 6 and 25 Hz 2nd order low-pass Butterworth filter. This had been done previously with similar data [41]. Six-degree-of-freedom joint powers at the knee and hip were calculated using the kinematic and kinetic data [42, 43].

To calculate ankle-foot power, we used a unified deformable (UD) power analysis. The analysis quantified everything distal to the shank as one deformable segment and captures the total power produced by the entire ankle-foot system [44]. This technique was used on both the prosthesis side and the contralateral side for consistency between limbs.

#### Statistical analysis

A linear mixed-effect model was used to determine the factors that affected the outcome variables (i.e., net metabolic power, prosthetic positive ankle-foot work, and ipsilateral positive hip work). Net metabolic power and joint works were normalized to the subject’s biological body mass. The analysis was a linear mixed-model with six-factors (random effect: participant; fixed effects: stiffness, load, stiffness squared, interaction of stiffness and load, interaction of stiffness squared and load). Previous studies involving exoskeletons or prostheses have seen both a linear [15] and quadratic [24, 45] relationship of stiffness with metabolic cost. Thus, we included both stiffness and stiffness squared terms in the model. Prosthetic ankle stiffness was calculated from inverse dynamics data for every participant/trial, and the load was the actual load percentage participants carried within the vest. All six factors were initially entered into the model, and stepwise elimination on the least significant variables was used until only the significant terms remained (*p* < 0.05). The remaining significant variables were included in the predictor equation for the outcome variables. The coefficients for these variables were reported, as well as the R^2^ value for the equation of the condition averages. This analysis was done for each outcome variable (MATLAB; MathWorks, Natick, MA).

In addition to the primary hypotheses, we performed several additional secondary analyses. A linear mixed-effect model was used to determine the factors that affected ipsilateral knee work, contralateral ankle-foot, knee and hip work, and perception data. A paired t-test was done between the metabolic cost measurements from the second to third day for each condition.

## Results

### Prosthetic ankle stiffness

**Fig. 2:**
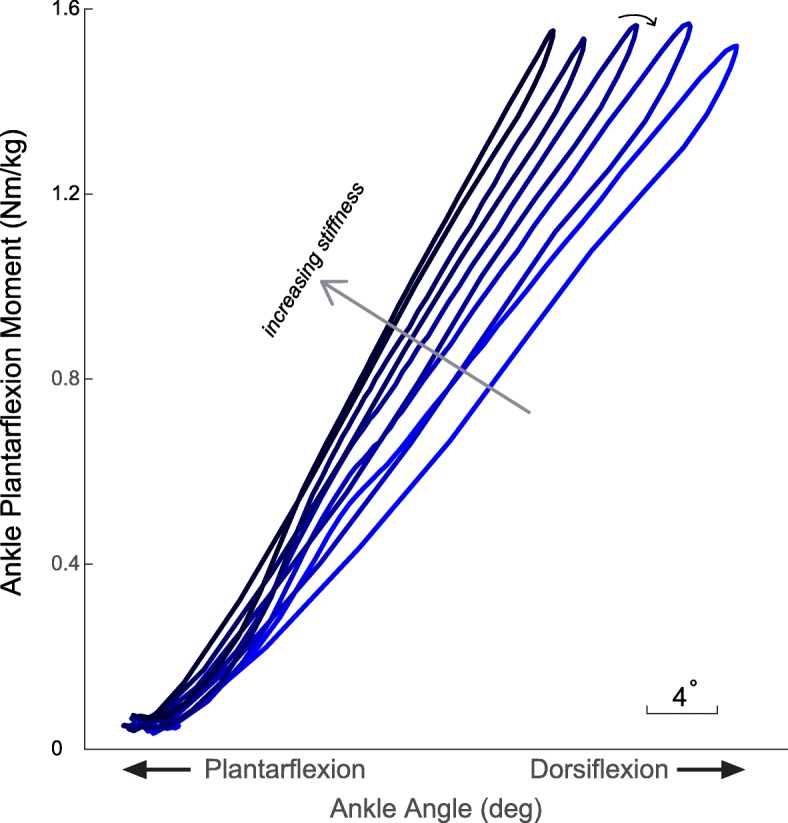
A representative participant’s moment-angle data**.** As the stiffness increased (indicated by the arrow direction), the slope of the moment-angle curve increased, confirming that the prosthetic emulator is capable of creating a range of stiffness profiles. The circular arrow indicates the direction of the moment-angle curve. We also note that the moment-angle curve also shows a slight hysteresis (i.e., net negative work), effectively simulating an unpowered and elastic prosthesis

The prosthetic emulator systematically changed stiffness consistent with the software input. This was shown by a representative participant’s prosthetic ankle moment-angle relationship for the five stiffness conditions at the normal walking conditions. As the input stiffness in the software increased, the slope of the measured moment-angle curve during walking increased (Fig. 2). The actual prosthetic ankle stiffness was calculated from experimentally-derived values during walking (via inverse dynamics) and compared to the stiffness value inputted (Additional file 2: Figure S2). While there were trials in which the actual stiffness values during walking were greater or lower than the prescribed input stiffness, the overall trend was consistent in that as the prescribed stiffness increased, the stiffness during the walking trials increased. The range of stiffness initially input into the software was between 0.0928 to 0.1392 Nm/deg/kg, but the actual range was larger since the experimentally-derived stiffness was not exactly the same as the prescribed input stiffness.

Additionally, we measured the stiffness calculated by the prosthesis software from the prosthesis load cell across all 3 days. (Additional file 3: Figure S3). We then did a t-test for each stiffness condition between days to determine if the stiffness varied within subjects among the 3 days of testing. We found that all differences were non-significant (*p* ≥ 0.0581) except the highest stiffness for the unloaded walking trial, with significant difference between days 2 and 3 (*p* = 0.0222).

### Joint angle and moments

**Fig. 3:**
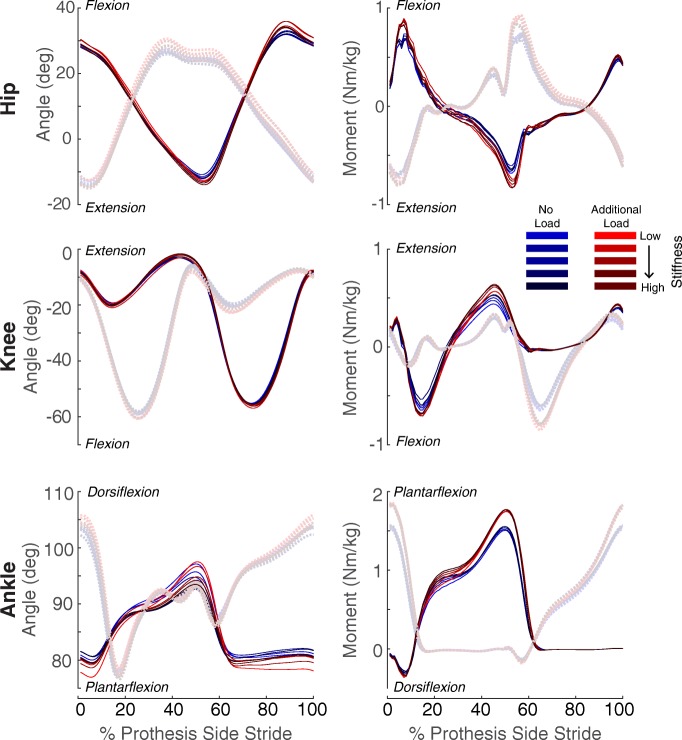
Time series (normalized to percentage of prosthesis-side stride cycle) of the average angle and moment data for the ankle, knee, and hip. The moment data is normalized to the participant’s body mass (not including the weighted vest). The solid lines are the prosthetic side, and the dashed lines are the contralateral side

Across the 10 walking conditions (five different stiffnesses and two load conditions), the ankle, knee, and hip had varying joint angles and moments (Fig. 3). Overall, the prosthetic ankle joint moment increased for the additional load conditions. Additionally, the ankle, knee, and hip had varying angular impulses across the 10 conditions (Additional file 7 Figure S7 and Additional file 8 Figure S8).

### Joint powers

**Fig. 4:**
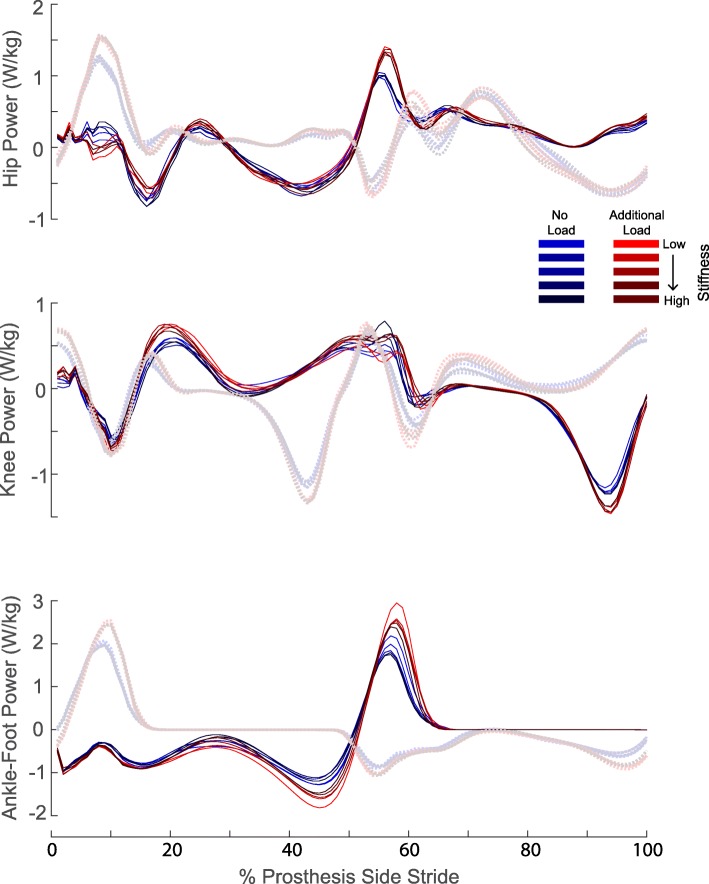
Time series (normalized to percentage of prosthesis-side stride cycle) of the average power for the ankle-foot, knee, and hip. The ankle-foot was calculated using the unified deformable segment analysis, and knee and hip were calculated using a 6 degree-of-freedom model. All power is normalized to the participant’s biological body mass. The solid lines are the prosthetic side and the dashed lines are the contralateral side

The total power of the ankle-foot, knee, and hip varied across the ten conditions for both the ipsilateral and contralateral side (Fig. 4). Overall, the prosthetic ankle-foot had an increase in peak power with load. The contralateral ankle-foot had an increasing trend with additional load (Fig. 4).

### Net metabolic power

**Fig. 5:**
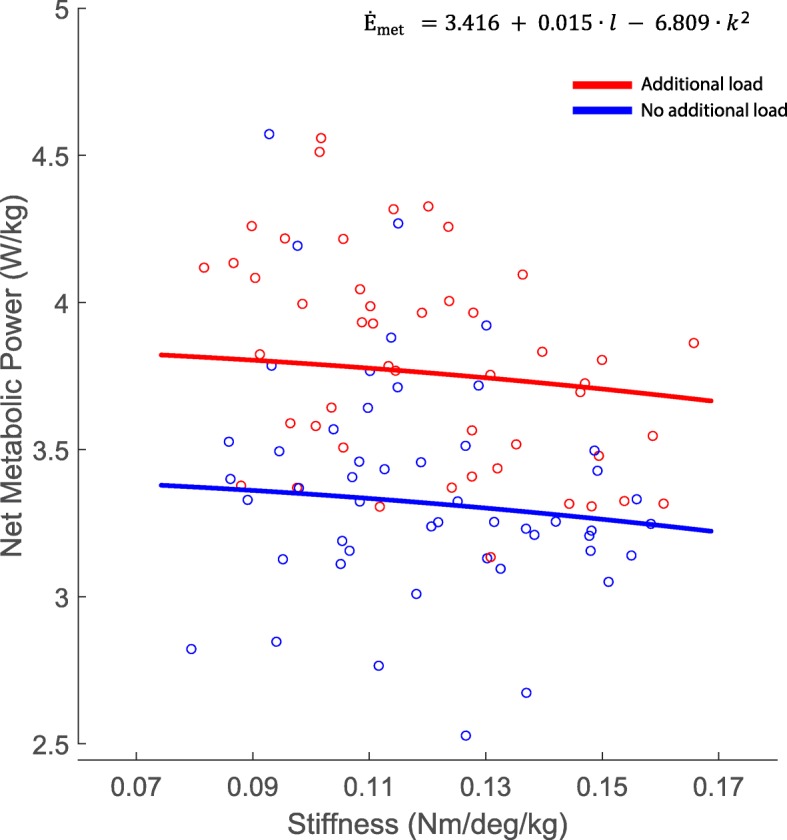
Net metabolic power (Ė_met_) was predicted by prosthetic ankle stiffness (*k*) and load (*l*). Net metabolic power was normalized to biological body mass. Each open circle is a participant’s data point. The blue line represents the equation at no load, and the red line represents the equation for the 30% additional load condition. ${\dot{\mathrm{E}}}_{met}=3.416+0.015\bullet l-6.809\bullet {k}^2$

Prosthetic ankle stiffness squared (k^2^, units: ${\left(\frac{Nm}{\mathit{\deg}\bullet kg}\right)}^2$) (*p* = 0.0356) and amount of load (*l,* units: *% body mass)*) (*p* < 0.001) were significant predictors of the net metabolic power (Ė_met)_ (adjusted R^2^ = 0.8480) (Fig. 5). Prosthetic ankle stiffness, the interaction of stiffness and load, and the interaction of stiffness squared and load were not significant, and thus were not included in the model. The model predicted the following equation: (Eq. 1)

1 $$ {\dot{\mathrm{E}}}_{met}\left(\frac{W}{kg}\right)=3.416+0.015\bullet l\kern0.5em -6.809\bullet {k}^2 $$

### Ipsilateral positive joint work

**Fig. 6:**
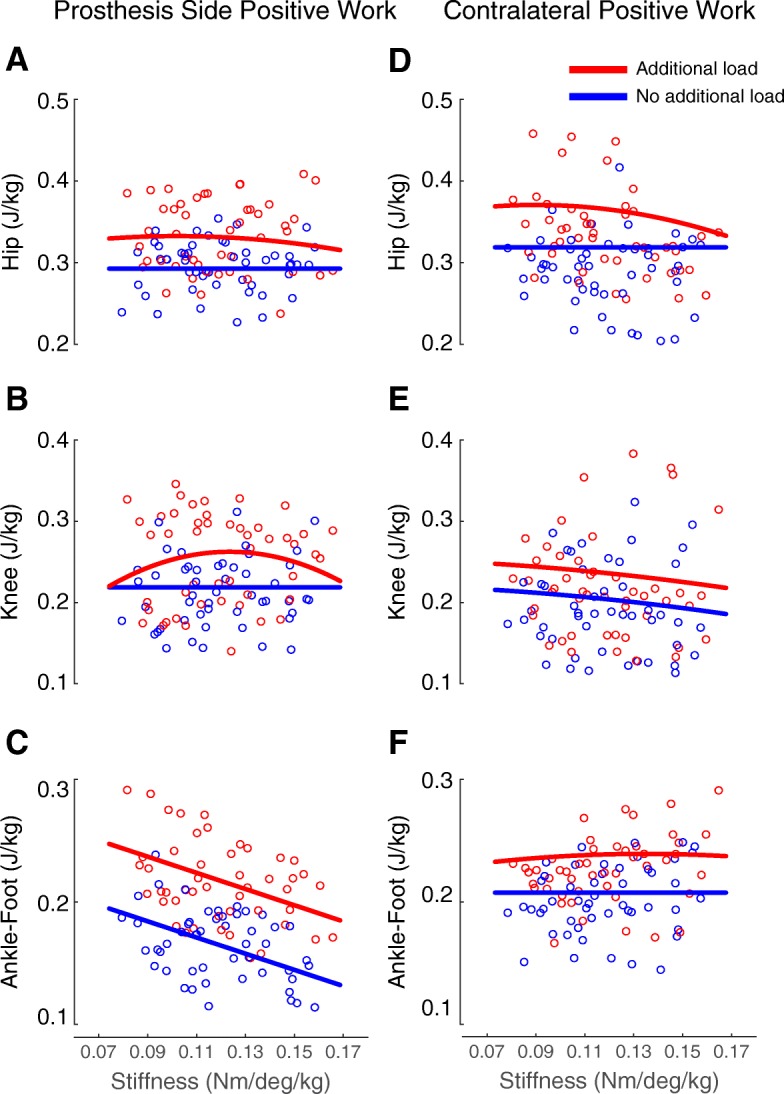
Results from the Linear Mixed Models for the ankle-foot, knee, and hip positive work on both the ipsilateral and contralateral side. Each open circle is a participant’s data point. The blue line represents the equation at no load, and the red line represents the equation for the 30% additional load condition. **a** Ipsilateral hip positive work (*W*_*IPS* _ *HIP*_) per stride was predicted by the interaction of stiffness (*k*) and load (*l*) as well as stiffness squared (*k*^*2*^) and load *W*_*IPS* _ *HIP*_ = 0.293 + 0.026 ∙ *kl* − 0.128 ∙ *k*^2^*l* (**b**) Ipsilateral knee positive work (*W*_*IPS* _ *KNEE*_) per stride was predicted by the load, the interaction of stiffness and load and the interaction of stiffness squared and load *W*_*IPS* _ *KNEE*_ = 0.219 − 0.007 ∙ *l* + 0.144 ∙ *kl* − 0.584 ∙ *k*^2^*l* (**c**) Prosthetic ankle-foot positive work (*W*_*PRO* _ *ANK* _ *FT*_) was predicted by stiffness and load *W*_*PRO* _ *ANK* _ *FT*_ = 0.244 + 0.002 ∙ *l* − 0.661 ∙ *k* (**d**) Contralateral hip work (*W*_*CON* _ *HIP*_) was predicted by the interaction of stiffness and load as well as the interaction of stiffness squared and load *W*_*CON* _ *HIP*_ = 0.319 + 0.038 ∙ *kl* − 0.208 ∙ *k*^2^*l* (**e**) Contralateral knee work (*W*_*CON* _ *KNEE*_) was predicted by stiffness squared and load. *W*_*CON* _ *KNEE*_ = 0.223 + 0.001 ∙ *l* − 1.293 ∙ *k*^2^ (**f**) Contralateral ankle foot positive work (*W*_*CON* _ *ANK* _ *FT*_) was predicted by the interaction of stiffness and load and the interaction of stiffness squared and load *W*_*CON* _ *ANK* _ *FT*_ = 0.208 + 0.016 ∙ *kl* − 0.057 ∙ *k*^2^*l*

Prosthetic ankle stiffness (k, units: $\frac{Nm}{\mathit{\deg}\bullet kg}$) (*p* < 0.001) and amount of load (*l*) (p < 0.001) were significant predictors of the prosthetic ankle-foot positive work (W_PRO_ANK_FT_) (adjusted R^2^ = 0.7995) (Fig. 6). Prosthetic ankle stiffness squared, the interaction of stiffness and load and the interaction of stiffness squared and load were not significant and were left out of the model. The resulting model equation was: (Eq. 2)

2 $$ {W}_{PRO\_\mathrm{A} NK\_ FT\kern0.5em }\left(\frac{J}{kg}\right)=0.244+0.002\bullet l-0.661\bullet k\kern0.5em $$

Ipsilateral hip positive work (W_IPS_HIP_) was related to the interaction of stiffness and load (p < 0.001) and interaction of the square of stiffness and load (*p* = 0.0023) (adjusted R^2^ = 0.6622) (Fig. 6). Prosthetic ankle stiffness, load, and stiffness squared were not significant and were removed from the model during the stepwise elimination. The model predicted the following equation for ipsilateral hip work: (Eq. 3)

3 $$ {W}_{IPS\_ HIP}\left(\frac{J}{kg}\right)\kern0.5em =0.293+0.026\bullet \mathrm{k}l\kern0.75em -0.128\bullet {k}^2l\kern0.5em $$

Load (*p* = 0.0399), the interaction of stiffness and load (*p* = 0.0157) and the interaction of stiffness squared and load (*p* = 0.0147) were significant predictors of ipsilateral knee positive work (W_IPS_KNEE_) (adjusted R^2^ = 0.8085) (Fig. 6). Prosthetic ankle stiffness and stiffness squared were not significant and were removed from the model. The model predicted the following equation for ipsilateral knee positive work: (Eq. 4)

4 $$ {W}_{IPS\_ KNEE}\left(\frac{J}{kg}\right)=0.219-0.007\bullet l+0.144\bullet kl-0.584\bullet {k}^2l\kern0.5em $$

### Contralateral positive joint work

The interaction of stiffness and load (*p* < 0.001), as well as the interaction of stiffness squared and load (*p* < 0.001) were significant predictors of contralateral hip positive work (W_CON_HIP_) (adjusted R^2^ = 0.8327) (Fig. 6). Prosthetic ankle stiffness, load, and stiffness squared were not significant and were removed from the final model. The equation from the model was: (Eq. 5)

5 $$ {W}_{CON\_ HIP}\left(\frac{J}{kg}\right)=0.319+0.038\bullet kl-0.208\bullet {k}^2l $$

Contralateral knee positive work (W_CON_KNEE_) was predicted by load (p < 0.001) and stiffness squared (*p* = 0.0084) (adjusted R^2^ = 0.9061) (Fig. 6). Prosthetic ankle stiffness, the interaction of stiffness and load, and the interaction of stiffness squared and load were not significant and excluded from the model. The resulting model was: (Eq. 6)

6 $$ {W}_{CON\_ KNEE}\left(\frac{J}{kg}\right)=0.223+0.001\bullet l-1.293\bullet {k}^2 $$

Contralateral ankle-foot positive work (W_CON_ANK_FT_) was predicted by the interaction of prosthetic ankle stiffness and load (p < 0.001) and the interaction of prosthetic ankle stiffness squared and load (*p* = 0.0326) (adjusted R^2^ = 0.8622) (Fig. 6). Stiffness, load, and stiffness squared were not significant and were excluded from the model. The resulting equation became: (Eq. 7)

7 $$ {W}_{CON\_ ANK\_ FT}\left(\frac{J}{kg}\right)=0.208+0.016\bullet kl-0.057\bullet {k}^2l $$

For additional analysis of the net metabolic power and joint power based on the categorical input stiffness, see Additional file 4: Figure S4, Additional file 5: Figure S5, and Additional file 6: Figure S6.

## Discussion

We used a robotic prosthetic emulator to simulate various ankle joint stiffnesses above and below a typical human ankle stiffness during different mechanical demands (e.g., with and without additional load). This experimental approach allowed us to vary the ankle joint stiffness while keeping every other parameter (e.g., foot length, mass, shape) constant on the device. We tested the hypothesis that the lowest stiffness would minimize metabolic cost for normal walking (no added load), and the stiffness that minimized metabolic cost during load carriage would be greater compared to the no load conditions. However, contrary to our hypothesis, the highest stiffness, out of the range tested, minimized metabolic energy for both walking conditions. We found no significant interaction between stiffness and load on metabolic energy cost.

One potential reason why our hypothesis was not supported is that we focused solely on the stiffness of the ankle joint and did not take into consideration other joints within the foot. The joints within the human foot are important and play a role in how the ankle-foot complex behaves during walking [46–51]. A study by Kern et al. found that the human midtarsal joint (i.e., arch) stiffness is about 2.5 times greater than the ankle joint, and both the midtarsal joint and ankle joint increase in stiffness when carrying additional loads [20]. Therefore, a higher stiffness might have been needed to minimize metabolic cost in order to incorporate the role of the midtarsal joint. A recent study involving an articulated toe and ankle prosthesis found that varying the toe joint stiffness affected whole-body mechanics just as much, if not more, than varying ankle joint stiffness [52]. The prosthesis used in our study did not have any articulations besides the ankle. Thus, in the absence of compliance within the foot arch or toes, the ankle stiffness may need to be stiffer than the typical human ankle stiffness in order to incorporate the functions of the foot structures as well.

The range of stiffness we tested could have influenced why the metabolic energy was minimized at the highest stiffness for both conditions. The range of input stiffness tested was from 0.0928 Nm/deg/kg to 0.1392 Nm/deg/kg, which is around the typical human ankle stiffness for normal walking, as well as walking with additional load [14, 18, 20, 34]. The actual stiffness range was larger since the experimentally-derived stiffness was not exactly the same as the prescribed input stiffness. It is possible that the range tested might have been too narrow to determine a different optimal stiffness for both load conditions. In comparison, Major et al. found that there was an 8% decrease in metabolic energy from their highest prosthetic stiffness (approximately 200% of our highest stiffness) to their lowest stiffness (approximately 80% of our lowest stiffness) in individuals with an amputation [15]. However, they did not test any stiffness values in the middle of the range, therefore it cannot be determined if there is a linear trend between the two points or if there is another trend that would appear if more values were tested.

From each participant’s highest stiffness to their lowest input stiffness, metabolic cost decreased by about 0.11 W/kg for the no load condition, or 3.2%, and 0.10 W/kg for the additional load conditions, or 2.57%. This difference would equate to the metabolic cost of transport of 0.01 J/Nm, which is less than the reported minimal detectable difference (0.022 J/Nm) using a portable metabolic system [53]. The study mentioned above was not using a prosthesis, which can influence the results of the minimal detectable difference. While the non-portable metabolic system we used for our study could be more accurate than the portable system, it is still likely that the difference in metabolic cost within the range of ankle stiffness we tested is relatively small. If the stiffness range tested was wider, it is possible that we could have seen a greater decrease in metabolic cost. Since there was a slight reduction in metabolic cost, we also wanted to see how participants’ perception of the difficulty of the condition related to this reduction.

We found that the participants’ perception of the difficulty of the condition was predicted by the interaction of load and stiffness (Additional file 9: Figure S9). Therefore, individuals did not perceive differences for the different stiffness values during the no additional load conditions, but they perceived that it was more unnatural to walk with increasing stiffness at the additional load conditions. This is the reverse of what was observed metabolically since their metabolic energy decreased as the stiffness increased for the additional load conditions. A previous study using a hip exoskeleton saw that participants did not always prefer the condition that was the most metabolically beneficial [54, 55]. Additionally, we performed a one-way repeated measures ANOVA to determine the relationship of order of trials with added mass on perception to determine if individuals perceived the later conditions as more difficult (i.e., potential fatigue effect). We found that there were no significant differences (*p* = 0.246), suggesting that subjects did not perceive later conditions as more difficult than others.

There was a great amount of variability between participants in terms of which condition minimized their metabolic energy expenditure. For each loading condition, we fit a quadratic regression to metabolic cost as a function of ankle stiffness and found the minimum of the curve, which was the stiffness that minimized metabolic cost within the range of stiffness tested. The average stiffness that minimized metabolic cost for the no load condition was 0.1166 ± 0.0247 Nm/deg/kg, and the average for the additional load condition was 0.1300 ± 0.0259 Nm/deg/kg. While the average stiffness that minimized metabolic cost increased with the additional load condition, the variability of each value was large in that one standard deviation is approximately 50% of the original range of stiffness tested in this study. This suggests that the stiffness that minimized metabolic cost might be better quantified on a per individual basis, since there is no single stiffness value that will minimize energy expenditure for all participants. A similar high participant to participant variability has been seen in exoskeletons and optimal timing profiles [55]. An approach like the ‘human in the loop’ optimization may be needed in order to find participant-specific parameters that will optimize the metabolic cost of walking, or any other variables, as seen in exoskeleton studies [56, 57]. The prosthesis that we used in this experiment is able to change stiffness on a step-to-step basis, therefore, it could be used with the “human in the loop” approach to vary stiffness until a metabolic minimum for each subject is reached. Such an iterative approach may be better suited to find a beneficial stiffness setting that accounts for the variability among individuals.

In partial support of our hypothesis, the lowest stiffness had the greatest amount of ankle-foot work for the no load conditions. Previous studies have seen similar trends where increasing prosthesis ankle joint stiffness decreases the amount of positive work in prosthetic devices [6, 16, 23, 24]. However, the stiffness condition that maximized prosthetic ankle-foot positive work did not correspond to the stiffness that minimized metabolic energy consumption. Instead, the stiffness that minimized prosthetic positive work (i.e., highest stiffness) corresponded to the stiffness that minimized metabolic cost. Ankle-foot work decreased by about 0.036 J/kg from the subject’s lowest stiffness to the highest stiffness, but this decrease only amounted to about a 3% decrease in metabolic cost. Previous studies that have modulated the amount of ankle work may suggest that a greater change in work may be needed to see a large change in metabolic energy [24, 31, 58]. Contrary to our findings, Caputo et al. used a powered prosthesis and found that for a work decrease of about 0.03 J/kg, there would be an increase of about 0.148 W/kg in metabolic cost [31]. Zelik et al. had about a 4.6 J difference in ankle push-off work (0.06 J/kg for a 75 kg person) from their lowest to highest stiffness but found no statistical significance between the metabolic cost at these two stiffness values [24]. The metabolic cost did decrease by about 7 to 8% from the highest to the medium stiffness, which had about a 2.6 J difference in push off work (0.035 J/kg for a 75 kg person) [24]. In a study with a commercially-available powered prosthesis, the prosthetist-chosen power setting was a mean ankle work of 0.11 ± 0.06 J/kg, but the best power setting for decreasing metabolic cost (by about 8.8% ± 4.6%) was 0.24 ± 0.07 J/kg [58]. Therefore, our differences in ankle-foot work between stiffness conditions may not have been large enough to influence the metabolic cost.

At the ipsilateral hip joint, our original hypothesis was that the lowest stiffness would minimize hip positive work, and a higher stiffness would minimize hip work when carrying additional loads. Our statistical model found that hip positive work did not significantly change between stiffness conditions for the no load condition. We found an interaction of both stiffness and load, as well as stiffness squared and load, which indicates that ipsilateral hip positive work varies as a function of both variables. Therefore, our hypothesis was partially supported, since the highest stiffness minimized hip positive work at the 30% load condition. This is important since many individuals with amputation experience an increase in metabolic cost, which can at least partly be attributed to their increase in hip positive work [9–11]. Upon further analysis, we found that both prosthetic ankle-foot positive work (*p* < 0.001) and ipsilateral hip positive work (p < 0.001) were significant predictors of metabolic cost (adjusted R^2^ = 0.7765). The equation was: ${\dot{\mathrm{E}}}_{met}=1.92+2.55\bullet {W}_{IPS\_ HIP}+4.30\bullet {W}_{PRO\_ ANK\_ FT}$. Therefore, as both the ankle-foot positive work and the ipsilateral hip positive work decreased, the metabolic cost decreased.

We also did a few secondary analyses to analyze the other joints to see if their behavior was influencing the net metabolic power outcomes. For the additional load conditions, the contralateral ankle-foot had more positive work in the higher stiffness conditions compared to the lower stiffness (Fig. 6), suggesting that it could be compensating for the reduced positive work of the prosthesis. The contralateral ankle-foot positive work did not significantly vary with stiffness for the no additional load conditions, which agrees with a previous study looking at prosthetic stiffness [24]. The ipsilateral and contralateral hip had similar behaviors, and they both varied as a function of both interaction terms.

This study had a few limitations. The prescribed prosthetic ankle stiffness was determined by approximating a single linear slope during the dorsiflexion phase based on the moment-angle relationship of human ankles [20]. A study by Shamaei et al. found that the human ankle is less stiff at the beginning of dorsiflexion compared to the end of the phase [13]. Thus, our prescribed ankle stiffness likely has a stiffer joint compared to the human ankle during early phases of dorsiflexion, which could explain the increase in ankle plantarflexion moment during the early stance phase compared to the contralateral ankle (Fig. 3). Additionally, most participants were inexperienced with walking with this prosthesis and the participants’ familiarity with load carriage was not assessed. To minimize the potential contributions of learning effects, we had each participant complete two training days before the testing day, which has been shown to be a sufficient amount of training with exoskeletons [59, 60]. A paired t-test was done for each condition between the first and second day, and the condition with 0% load, and the med-high stiffness value was significantly different (*p* = 0.0448). However, all other conditions were not significantly different (*p* ≥ 0.14) (Additional file 10: Figure S10). When the same paired t-test was done for each condition between the second and third day, all conditions were not significantly different between the 2 days (*p* ≥ 0.11). This may suggest that the trends between metabolic cost were similar between the second and third day and no additional changes in metabolic cost happened due to learning.

Another limitation is the generalizability of these findings. The participant recruitment was limited in that there were two sizes of lift shoes used, and there was a specific weight limit for the prosthesis. Because of these criteria, we had a restricted range of participants based on shoe size and body mass. While this increases the internal validity of the study, the findings of our study are less generalizable. In addition, using a simulator boot with the prosthesis makes these results less generalizable to individuals with amputation. There have been a variety of studies that have used emulator or simulator boots [24, 31, 33, 52]. Some studies have shown similar results between healthy controls and individuals with amputation [24], while others have shown differing results [24, 31, 35]. It is currently unclear whether the findings of our study could translate to individuals with amputation, and our findings should be verified in individuals with amputation before informing the design of prosthesis. Having healthy controls walk on simulator boots with the prosthesis helps to eliminate the amputation-specific variability including residual limb length, amputation type, amputation surgery and socket interface as well as the common comorbidities with amputations such as residual limb pain, osteoarthritic pain and scoliosis [24, 52]. Additionally, using simulator boots can show how healthy humans adapt to different toe and ankle properties [52]. The added mass of the simulator boot and added leg length can influence the results. The average metabolic cost for our study while walking with this emulator without additional load was 3.3 W/kg. Another study using a similar prosthetic emulator found that the average metabolic cost of healthy individuals walking with this emulator was 3.6 W/kg, while these same individuals had an average metabolic cost of 2.7 ± 0.37 W/kg while walking with their normal shoes [31]. This suggests that walking with this emulator could increase metabolic cost. However, our experimental protocol was a within-subjects design so the effect should be approximately the same for each condition when comparing between the conditions for each participant.

The purpose of this study was to understand the importance of changing ankle stiffness during different locomotor tasks. While human ankle’s ability to modulate joint stiffness is well documented [14, 17, 18, 20], our study found that the magnitude of changes in human ankle stiffness seen between normal (no load) walking and load carriage conditions [18, 20] likely leads to a small change in metabolic cost. Such findings may indicate that the functional importance of human ankle’s ability to modulate stiffness may involve factors besides minimizing metabolic cost across various locomotion tasks, such as maximizing stability or reducing fall risks.

## Conclusion

Our study revealed that a stiffness higher than the typical human ankle can decrease metabolic energy at both normal walking and walking with additional loads. Additionally, we found no significant interaction between stiffness and load carriage on metabolic cost, which may suggest the modulating ankle stiffness comparable to levels of how the human ankle modulates stiffness is not likely to have a metabolic benefit. Future studies looking at a wider stiffness range or using human-in-the-loop optimization can be done to further solidify if there is an ‘optimal’ stiffness that can be determined when individuals walk under various mechanical demands.

## Supplementary information

**Additional file 1: Figure S1.** Moment-Angle Relationship from the HuMoTech prosthesis emulator MATLAB/Simulink code. Two moment and angle values-pairs were entered into the software to define a linear slope and created the desired moment-angle relationship (indicated by the circles outlined in black). The first pair of points were always at a plantarflexion moment of 0 Nm and at 0 degrees dorsiflexion. The second pair of points were a condition-specific non-zero dorsiflexion value and plantarflexion moment value. Taking the slope of the best fit line to the moment-angle curve gave an estimate of the ankle dorsiflexion stiffness. The figure above shows a representative moment-angle curve from ‘low’ stiffness (blue) and ‘high’ stiffness (green) conditions.

**Additional file 2: Figure S2.** Input stiffness versus stiffness from inverse dynamics. The stiffness values input into the prosthesis software versus the stiffness values calculated from the inverse dynamics. Each dot represents one participant’s data, and the five stiffness values for each participant are connected with a solid line. The blue lines represent the no load conditions, and the red lines represent the additional load conditions. The black diagonal line shows where the two values would be equal. The black dashed line is a best fit line to all data. This graph shows that while the stiffness that is inputted is not always the exact value found from inverse dynamics, there is still a general trend for increasing stiffness when an increased stiffness is inputted.

**Additional file 3: Figure S3.** Prosthesis stiffness for each condition across all 3 days**.** The stiffness values calculated in the prosthesis software from the load cell on the prosthesis across the three days of testing. We did a t-test for each stiffness condition between days to determine if the stiffness varied within subjects among the three days of testing. We found that all differences were insignificant (*p* ≥ 0.0581) except the highest stiffness for the unloaded walking trial, with a significant difference between days 2 and 3 (*p* = 0.0222).

**Additional file 4: Figure S4.** Metabolic cost for each input stiffness condition. All values are normalized to biological body mass. Blue bars represent the no load conditions, and the red bars represent the additional load conditions. As the colors get darker for both loading conditions, the stiffness values are increasing.

**Additional file 5: Figure S5**. Prosthetic ankle-foot, and ipsilateral knee and hip work per stride for each condition. All values are normalized to biological body mass. Blue bars represent the no additional load, and red bars represent the additional load conditions. As the colors get darker, the stiffness values are increasing.

**Additional file 6: Figure S6.** Contralateral ankle-foot, knee, and hip work per stride for each condition. All values are normalized to biological body mass. Blue bars represent the no additional load, and red bars represent the additional load conditions. As the colors get darker, the stiffness values are increasing.

**Additional file 7 Figure S7.** Ipsilateral ankle, knee, and hip angular impulse for each condition. All values are normalized to biological body mass. Blue bars represent the no additional load, and red bars represent the additional load conditions. As the colors get darker, the stiffness values are increasing.

**Additional file 8: Figure S8.** Contralateral ankle, knee, and hip angular impulse for each condition**.** All values are normalized to biological body mass. Blue bars represent the no additional load, and red bars represent the additional load conditions. As the colors get darker, the stiffness values are increasing.

**Additional file 9: Figure S9.** Participants’ perception of each conditions’ difficulty, compared to the actual stiffness of the condition. Each dot represents each participant’s individual data, and the solid lines are the predicted equation. The interaction of stiffness and load were significant predictors of the participant’s perception. *Perception* =  − 1.91 − 0.23 ∙ *kl* Therefore, for the no load condition, participant’s did not perceive any difference in difficulty, but for the additional load conditions, the conditions seemed more difficult as stiffness increased.

**Additional file 10: Figure S10.** Metabolic Cost for each condition across all 3 days. A paired t-test between the second and third day metabolic cost shows that there was no differences between the metabolic cost values for these days (*p* < 0.05).

## Data Availability

Please contact the authors for data requests

## Abbreviations

Ė_met_: net metabolic power (W/kg)
k: stiffness $$ \left(\frac{Nm}{\mathit{\deg}\cdot kg}\right) $$
*l*: amount of load (% body mass)
UD: Unified deformable
W_CON_ANK_FT_: contralateral ankle positive work (J/kg)
W_CON_HIP_: contralateral hip positive work (J/kg)
W_CON_KNEE_: contralateral knee positive work (J/kg)
W_IPS_HIP_: ipsilateral hip positive work (J/kg)
W_IPS_KNEE_: ipsilateral knee positive work (J/kg)
W_PRO_ANK_FT_: prosthetic ankle-foot positive work (J/kg)

## References

[1] Neptune RR, Kautz SA, Zajac FE (2001). Contributions of the individual ankle plantar flexors to support, forward progression and swing initiation during walking. J Biomech.

[2] Huang TP, Shorter KA, Adamczyk PG, Kuo AD (2015). Mechanical and energetic consequences of reduced ankle plantar-flexion in human walking. J Exp Biol.

[3] Zelik KE, Kuo AD (2010). Human walking isn’t all hard work: evidence of soft tissue contributions to energy dissipation and return. J Exp Biol.

[4] DeVita P, Hortobagyi T (2000). Age causes a redistribution of joint torques and powers during gait. J Appl Physiol.

[5] Nadeau S, Gravel D, Arsenault AB, Bourbonnais D (1999). Plantarflexor weakness as a limiting factor of gait speed in stroke subjects and the compensating role of hip flexors. Clin Biomech.

[6] Fey NP, Klute GK, Neptune RR (2011). The influence of energy storage and return foot stiffness on walking mechanics and muscle activity in below-knee amputees. Clin Biomech.

[7] Waters RL, Mulroy S (1999). The energy expenditure of normal and pathologic gait. Gait Posture.

[8] Sadeghi H, Allard P, Duhaime M (2001). Muscle power compensatory mechanisms in below-knee amputee gait. Am J Phys Med Rehabil.

[9] Russell Esposito E, Whitehead JMA, Wilken JM (2016). Step-to-step transition work during level and inclined walking using passive and powered ankle-foot prostheses. Prosthetics Orthot Int.

[10] Houdijk H, Pollmann E, Groenewold M, Wiggerts H, Polomski W (2009). The energy cost for the step-to-step transition in amputee walking. Gait Posture..

[11] Schmalz T (2002). Blumentritt S, Jarasch R. energy expenditure and biomechanical characteristics of lower limb amputee gait: the influence of prosthetic alignment and different prosthetic components. Gait Posture..

[12] Hansen AH, Childress DS, Miff SC, Gard SA, Mesplay KP (2004). The human ankle during walking: implications for design of biomimetic ankle prostheses. J Biomech.

[13] Shamaei K, Sawicki GS, Dollar AM. Estimation of Quasi-Stiffness and Propulsive Work of the Human Ankle in the Stance Phase of Walking. PLoS One. 2013;8(3). 10.1371/journal.pone.0059935 PMC3605342 23555839

[14] Safaeepour Z, Esteki A, Ghomshe FT, Abu Osman NA (2014). Quantitative analysis of human ankle characteristics at different gait phases and speeds for utilizing in ankle-foot prosthetic design. Biomed Eng Online.

[15] Major MJ, Twiste M, Kenney LPJ, Howard D (2014). The effects of prosthetic ankle stiffness on ankle and knee kinematics, prosthetic limb loading, and net metabolic cost of trans-tibial amputee gait. Clin Biomech.

[16] Shell CE, Segal AD, Klute GK, Neptune RR (2017). The effects of prosthetic foot stiffness on transtibial amputee walking mechanics and balance control during turning. Clin Biomech.

[17] Collins JD, Arch ES, Crenshaw JR, Bernhardt KA, Khosla S, Amin S (2018). Net ankle quasi-stiffness is influenced by walking speed but not age for older adult women. Gait Posture..

[18] Shamaei K, Cenciarini M, Dollar AM. On the mechanics of the knee during the stance phase of the gait On the Mechanics of the Knee during the Stance Phase of the Gait. In: 33rd Annual International Conference of the IEEE EMBS. 2011. p. 8135–8140. 10.1109/IEMBS.2011.6092007 22256230

[19] Argunsah Bayram H, Bayram MB (2018). Dynamic functional stiffness index of the ankle joint during daily living. J Foot Ankle Surg.

[20] Kern AM, Papachatzis N, Patterson JM, Bruening DA, Takahashi KZ. Ankle and midtarsal joint quasi-stiffness during walking with added mass. PeerJ. 2019;7:7:e7487. 10.7717/peerj.7487 PMC6754976 31579566

[21] Sawicki GS, Lewis CL, Ferris DP (2009). It pays to have a spring in your step. Exerc Sport Sci Rev.

[22] Adamczyk PG, Roland M, Hahn ME (2017). Sensitivity of biomechanical outcomes to independent variations of hindfoot and forefoot stiffness in foot prostheses. Hum Mov Sci.

[23] Fey NP, Klute GK, Neptune RR (2013). Altering prosthetic foot stiffness influences foot and muscle function during below-knee amputee walking: a modeling and simulation analysis. J Biomech.

[24] Zelik KE, Collins SH, Adamczyk PG, Segal AD, Klute GK, Morgenroth DC (2011). Systematic variation of prosthetic foot spring affects center-of-mass mechanics and metabolic cost during walking. IEEE Trans Neural Syst Rehabil Eng..

[25] Doyle SS, Lemaire ED, Besemann M, Dudek NL (2014). Changes to level ground transtibial amputee gait with a weighted backpack. Clin Biomech.

[26] Schnall BL, Wolf EJ, Bell JC, Gambel J, Bensel CK (2012). Metabolic analysis of male servicemembers with transtibial amputations carrying military loads. J Rehabil Res Dev.

[27] Shepherd MK, Rouse EJ (2017). The VSPA foot a quasi-passive ankle-foot. IEEE Trans Neural Syst Rehabil Eng..

[28] Glanzer EM, Adamczyk PG (2018). Design and validation of a semi-active variable stiffness foot prosthesis. IEEE Trans Neural Syst Rehabil Eng.

[29] Huang TP, Kuo AD (2014). Mechanics and energetics of load carriage during human walking. J Exp Biol.

[30] Adamczyk PG, Collins SH, Kuo AD (2006). The advantages of a rolling foot in human walking. J Exp Biol.

[31] Caputo JM, Collins SH (2014). Prosthetic ankle push-off work reduces metabolic rate but not collision work in non-amputee walking. Sci Rep.

[32] Zelik KE, Honert EC (2018). Ankle and foot power in gait analysis: implications for science, technology and clinical assessment. J Biomech.

[33] Malcolm P, Quesada RE, Caputo JM, Collins SH. The influence of push-off timing in a robotic ankle-foot prosthesis on the energetics and mechanics of walking. J Neuroeng Rehabil. 2015;12(1). 10.1186/s12984-015-0014-8 PMC4404655 25889201

[34] Mager F, Richards J, Hennies M, Dotzel E, Chohan A, Mbuli A (2018). Determination of ankle and metatarsophalangeal stiffness during walking and jogging. J Appl Biomech.

[35] Quesada RE, Caputo JM, Collins SH (2016). Increasing ankle push-off work with a powered prosthesis does not necessarily reduce metabolic rate for transtibial amputees. J Biomech.

[36] Grenier JG, Peyrot N, Castells J, Oullion R, Messonnier L, Morin JB (2012). Energy cost and mechanical work of walking during load carriage in soldiers. Med Sci Sports Exerc.

[37] Griffin TM, Roberts TJ, Kram R (2003). Metabolic cost of generating muscular force in human walking: insights from load-carrying and speed experiments. J Appl Physiol.

[38] Wilken JM, Rodriguez KM, Brawner M, Darter BJ (2012). Reliability and minimal detectible change values for gait kinematics and kinetics in healthy adults. Gait Posture..

[39] Crouter SE, Antczak A, Hudak JR, DellaValle DM, Haas JD (2006). Accuracy and reliability of the ParvoMedics TrueOne 2400 and MedGraphics VO2000 metabolic systems. Eur J Appl Physiol.

[40] Brockway JM (1987). Derivation of formulae used to calculate energy expenditure in man. Hum Nutr Clin Nutr.

[41] Takahashi KZ, Gross MT, Van Werkhoven H, Piazza SJ, Sawicki GS (2016). Adding stiffness to the foot modulates soleus force-velocity behaviour during human walking. Sci Rep.

[42] Zelik KE, Takahashi KZ, Sawicki GS (2015). Six degree-of-freedom analysis of hip, knee, ankle and foot provides updated understanding of biomechanical work during human walking. J Exp Biol.

[43] Buczek FL, Kepple TM, Siegel KL, Stanhope SJ (1994). Translational and rotational joint power terms in a six degree-of-freedom model of the normal ankle complex. J Biomech.

[44] Takahashi KZ, Kepple TM, Stanhope SJ (2012). A unified deformable (UD) segment model for quantifying total power of anatomical and prosthetic below-knee structures during stance in gait. J Biomech.

[45] Collins SH, Wiggin MB, Sawicki GS (2015). Reducing the energy cost of human walking using an unpowered exoskeleton. Nature..

[46] Takahashi KZ, Worster K, Bruening DA (2017). Energy neutral: the human foot and ankle subsections combine to produce near zero net mechanical work during walking. Sci Rep.

[47] Takahashi KZ, Stanhope SJ (2013). Mechanical energy profiles of the combined ankle-foot system in normal gait: insights for prosthetic designs. Gait Posture..

[48] Rolian C, Lieberman DE, Hamill J, Scott JW, Werbel W (2009). Walking, running and the evolution of short toes in humans. J Exp Biol.

[49] Ker RF, Bennett MB, Bibby SR, Kester RC, Alexander RM (1987). The spring in the arch of the human foot. Nature..

[50] MacWilliams BA, Cowley M, Nicholson DE (2003). Foot kinematics and kinetics during adolescent gait. Gait Posture..

[51] Farris DJ, Kelly LA, Cresswell AG, Lichtwark GA (2019). The functional importance of human foot muscles for bipedal locomotion. Proc Natl Acad Sci.

[52] Honert EC, Bastas G, Zelik KE. Effect of toe joint stiffness and toe shape on walking biomechanics. Bioinspiration and Biomimetics. 2018;13(6):aadf46. 10.1088/1748-3190/aadf46 PMC8777388 30187893

[53] Davidson A, Gardinier ES, Gates DH (2016). Within and between-day reliability of energetic cost measures during treadmill walking. Cogent Eng.

[54] Young AJ, Gannon H, Ferris DP (2017). A biomechanical comparison of proportional electromyography control to biological torque control using a powered hip exoskeleton. Front Bioeng Biotechnol.

[55] Young AJ, Foss J, Gannon H, Ferris DP (2017). Influence of power delivery timing on the energetics and biomechanics of humans wearing a hip exoskeleton. Front Bioeng Biotechnol..

[56] Zhang J, Fiers P, Witte KA, Jackson RW, Poggensee KL, Atkeson CG (2017). Human-in-the-loop optimiziation of exoskeleton assistance during walking. Sci Robot.

[57] Ding Y, Kim M, Kuindersma S, Walsh CJ (2018). Human-in-the-loop optimization of hip assistance with a soft exosuit during walking. Sci Robot..

[58] Ingraham KA, Choi H, Gardinier ES, Remy CD, Gates DH (2018). Choosing appropriate prosthetic ankle work to reduce the metabolic cost of individuals with transtibial amputation. Sci Rep.

[59] Koller JR, Jacobs DA, Ferris DP, Remy CD (2015). Learning to walk with an adaptive gain proportional myoelectric controller for a robotic ankle exoskeleton. J Neuroeng Rehabil.

[60] Panizzolo FA, Freisinger GM, Karavas N, Eckert-Erdheim AM, Siviy C, Long A (2019). Metabolic cost adaptations during training with a soft exosuit assisting the hip joint. Sci Rep.

