# Supplementary figures and images for: The effects of ankle stiffness on mechanics and energetics of walking with added loads: a prosthetic emulator study
Source: J Neuroeng Rehabil. 2019 Nov 21;16:148. doi: 10.1186/s12984-019-0621-x (PMC6873504; doi:10.1186/s12984-019-0621-x)

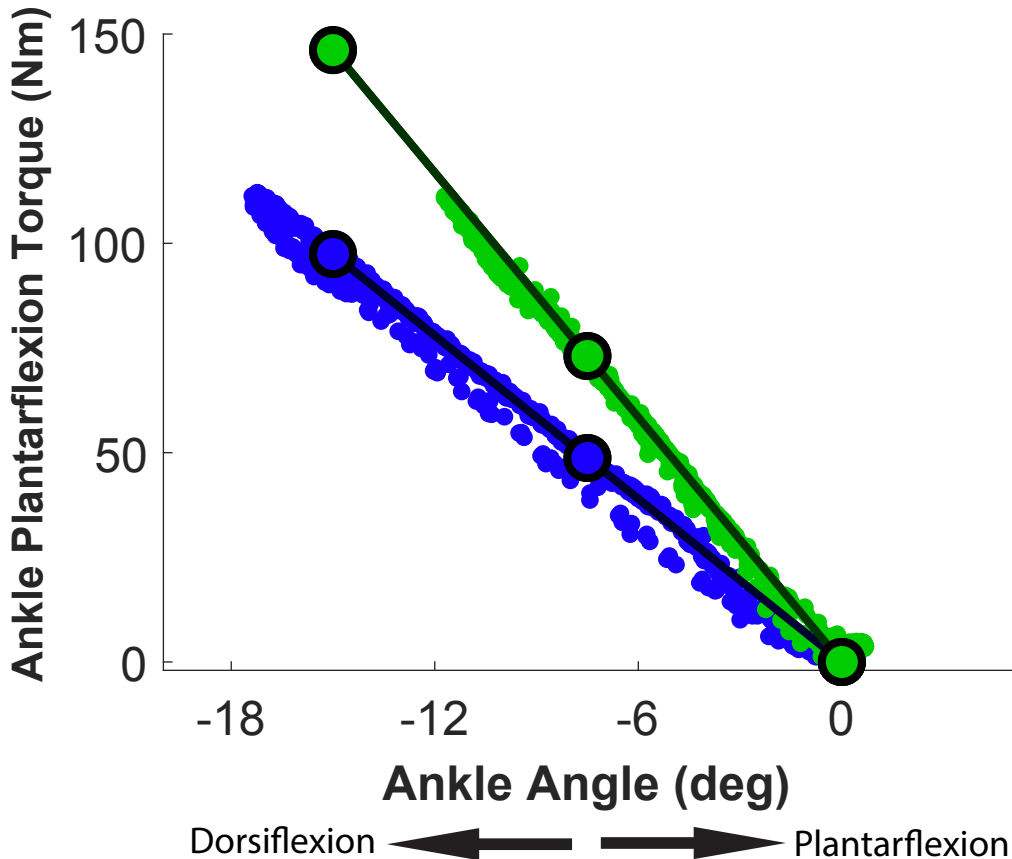

Supplement: Supplementary file 1 — Additional file 1: Figure S1. Moment-Angle Relationship from the HuMoTech prosthesis emulator MATLAB/Simulink code. Two moment and angle values-pairs were entered into the software to define a linear slope and created the desired moment-angle relationship (indicated by the circles outlined in black). The first pair of points were always at a plantarflexion moment of 0 Nm and at 0 degrees dorsiflexion. The second pair of points were a condition-specific non-zero dorsiflexion value and plantarflexion moment value. Taking the slope of the best fit line to the moment-angle curve gave an estimate of the ankle dorsiflexion stiffness. The figure above shows a representative moment-angle curve from ‘low’ stiffness (blue) and ‘high’ stiffness (green) conditions. [file 12984_2019_621_MOESM1_ESM.pdf]

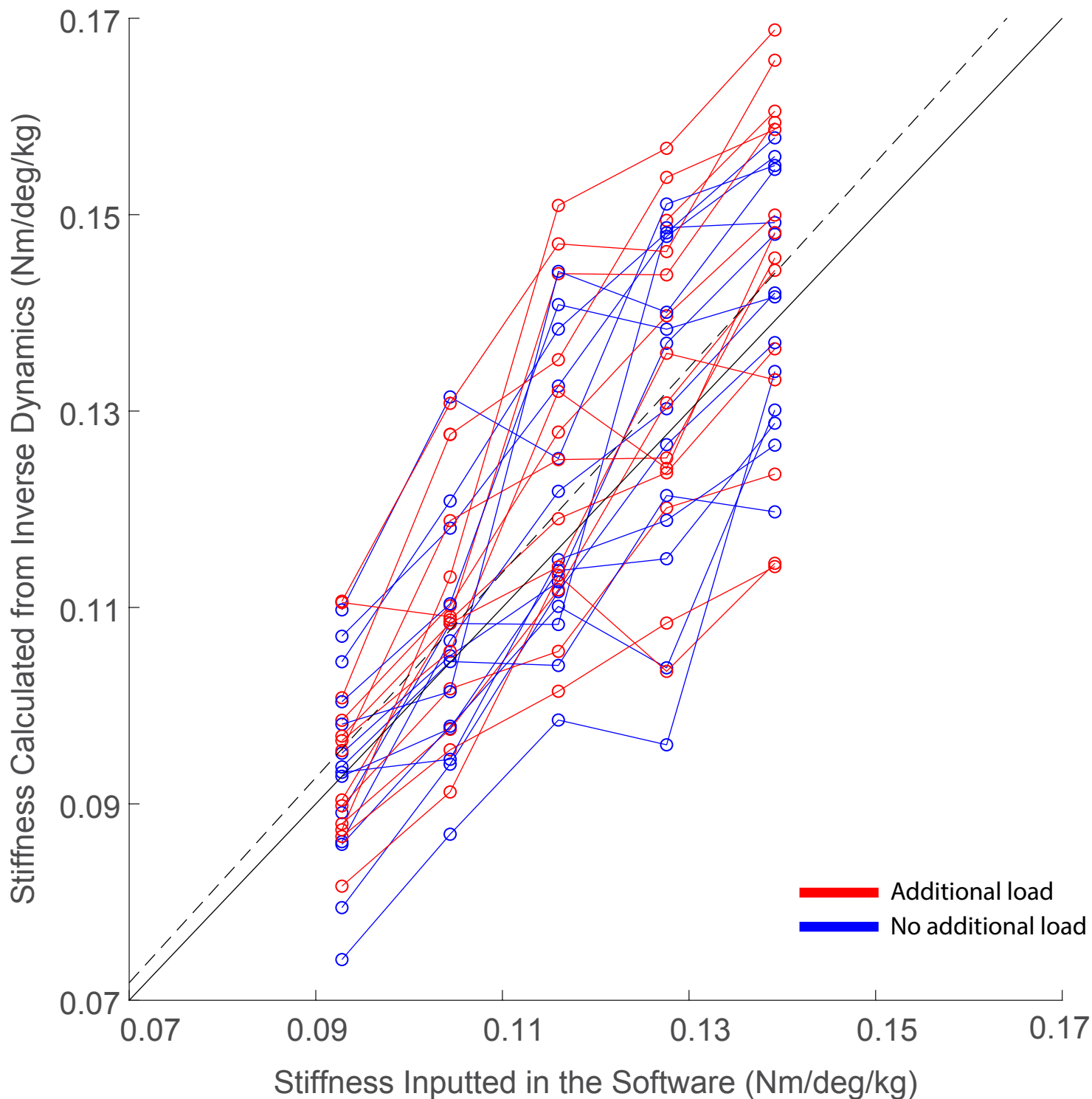

Supplement: Supplementary file 2 — Additional file 2: Figure S2. Input stiffness versus stiffness from inverse dynamics. The stiffness values input into the prosthesis software versus the stiffness values calculated from the inverse dynamics. Each dot represents one participant’s data, and the five stiffness values for each participant are connected with a solid line. The blue lines represent the no load conditions, and the red lines represent the additional load conditions. The black diagonal line shows where the two values would be equal. The black dashed line is a best fit line to all data. This graph shows that while the stiffness that is inputted is not always the exact value found from inverse dynamics, there is still a general trend for increasing stiffness when an increased stiffness is inputted. [file 12984_2019_621_MOESM2_ESM.pdf]

## No Additional Load

## Additional Load

Low  
Stiffness

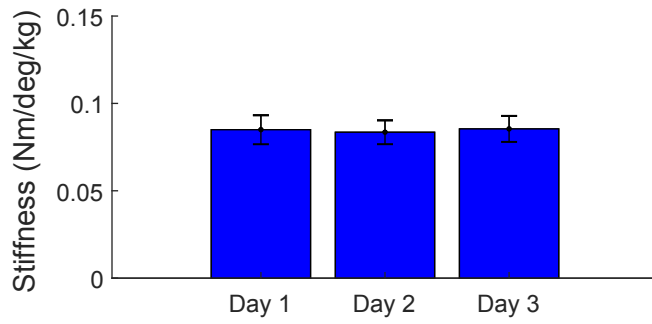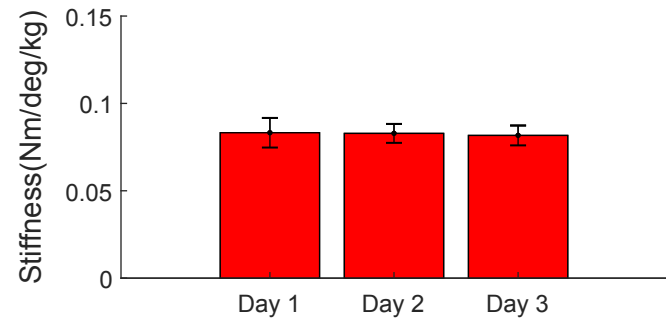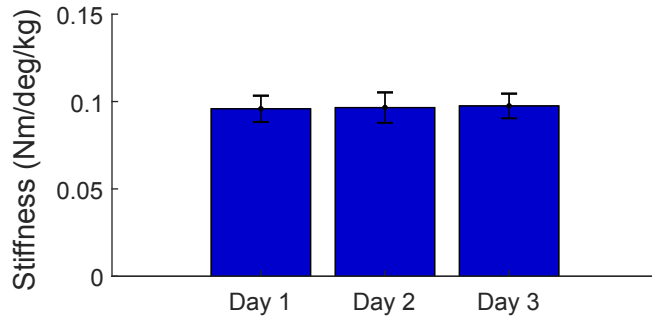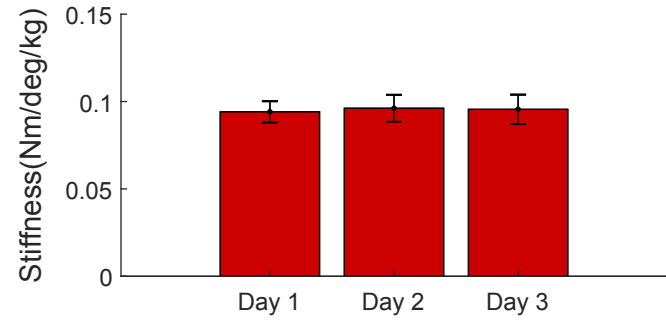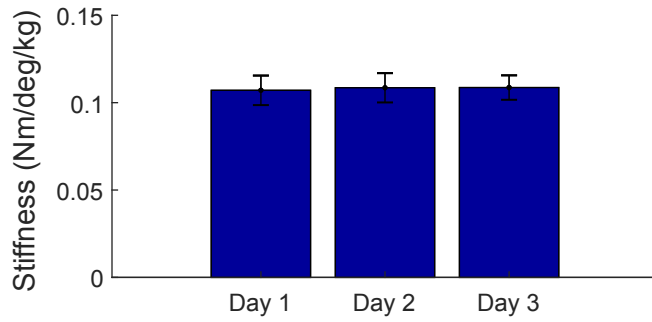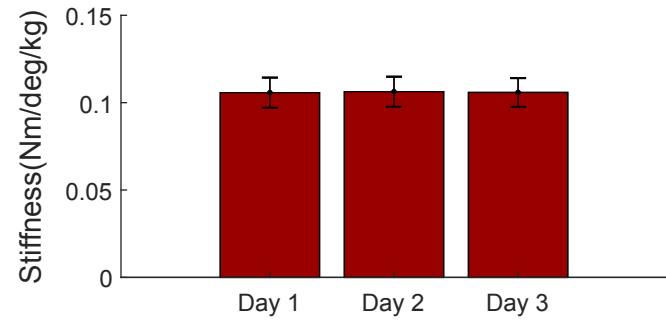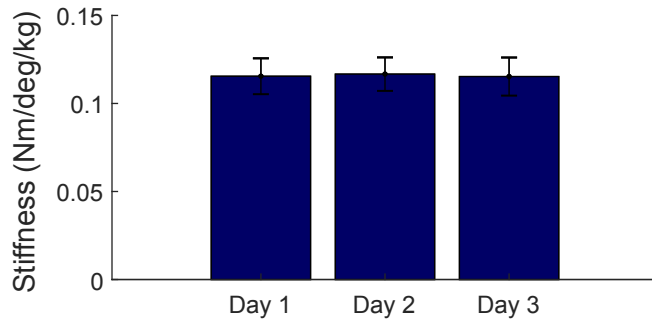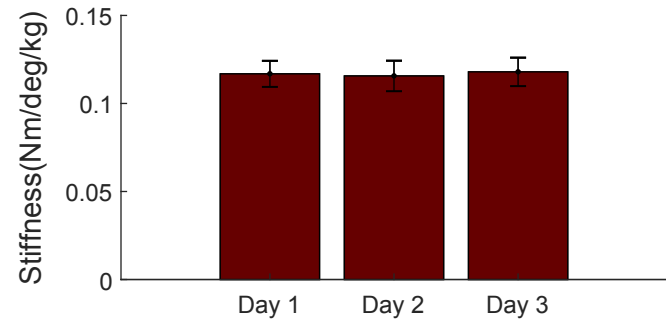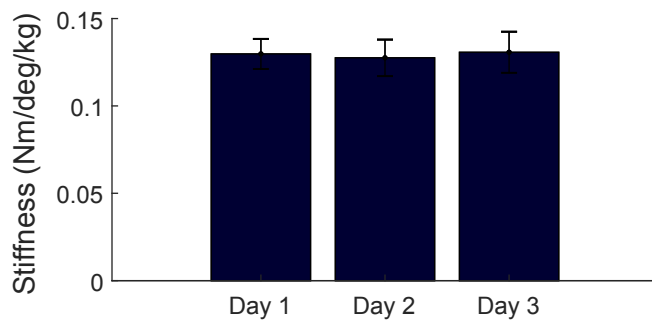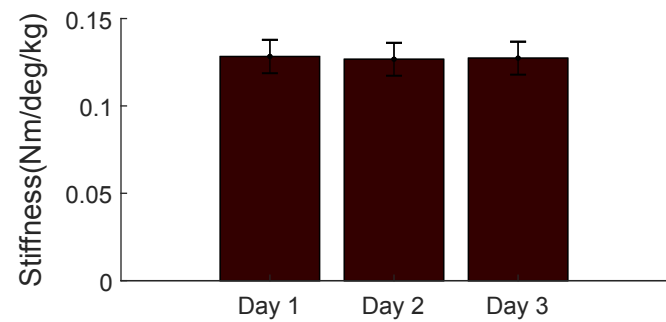

High  
Stiffness

Supplement: Supplementary file 3 — Additional file 3: Figure S3. Prosthesis stiffness for each condition across all 3 days. The stiffness values calculated in the prosthesis software from the load cell on the prosthesis across the three days of testing. We did a t-test for each stiffness condition between days to determine if the stiffness varied within subjects among the three days of testing. We found that all differences were insignificant (p ≥ 0.0581) except the highest stiffness for the unloaded walking trial, with a significant difference between days 2 and 3 (p = 0.0222). [file 12984_2019_621_MOESM3_ESM.pdf]

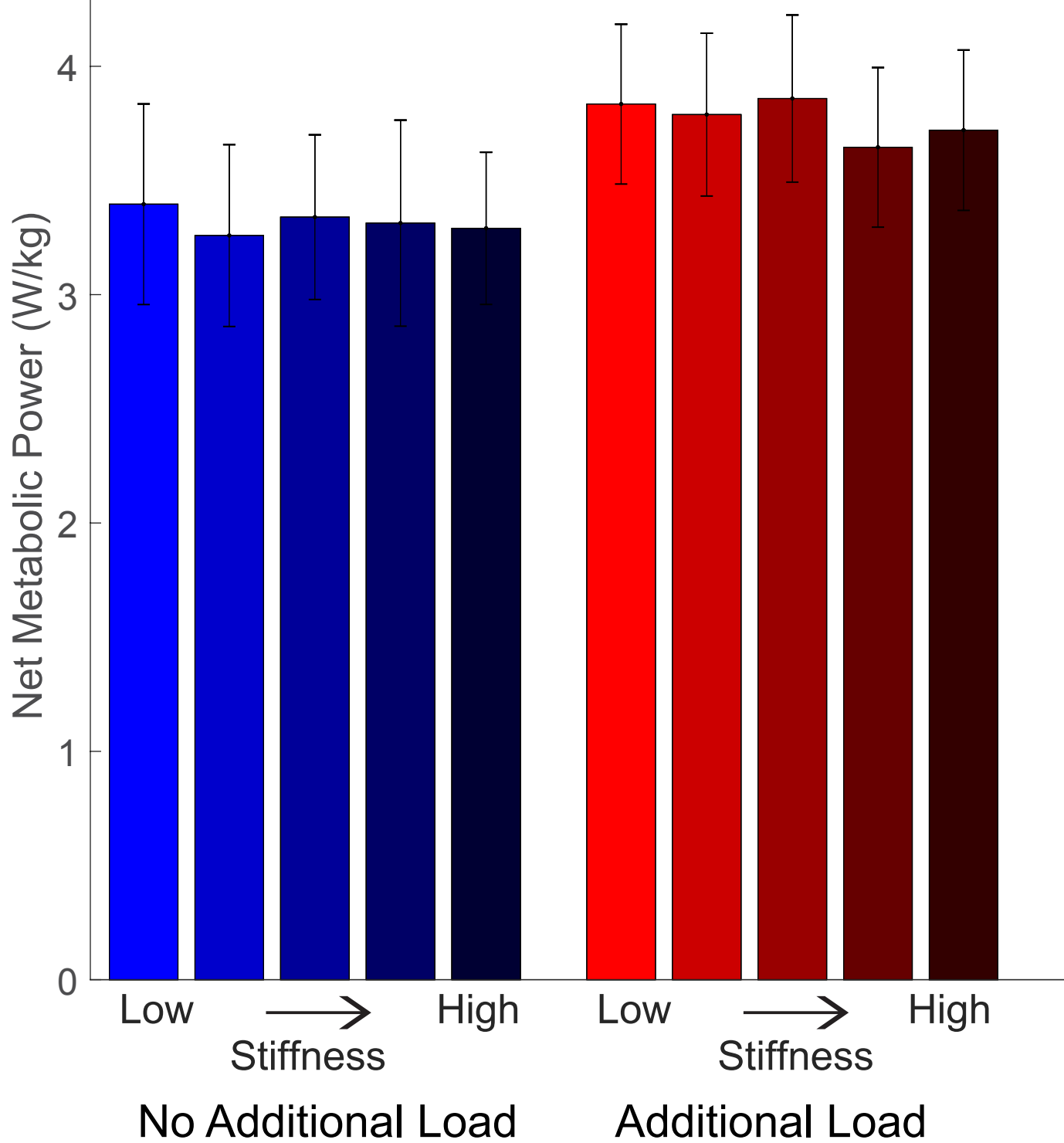

Supplement: Supplementary file 4 — Additional file 4: Figure S4. Metabolic cost for each input stiffness condition. All values are normalized to biological body mass. Blue bars represent the no load conditions, and the red bars represent the additional load conditions. As the colors get darker for both loading conditions, the stiffness values are increasing. [file 12984_2019_621_MOESM4_ESM.pdf]

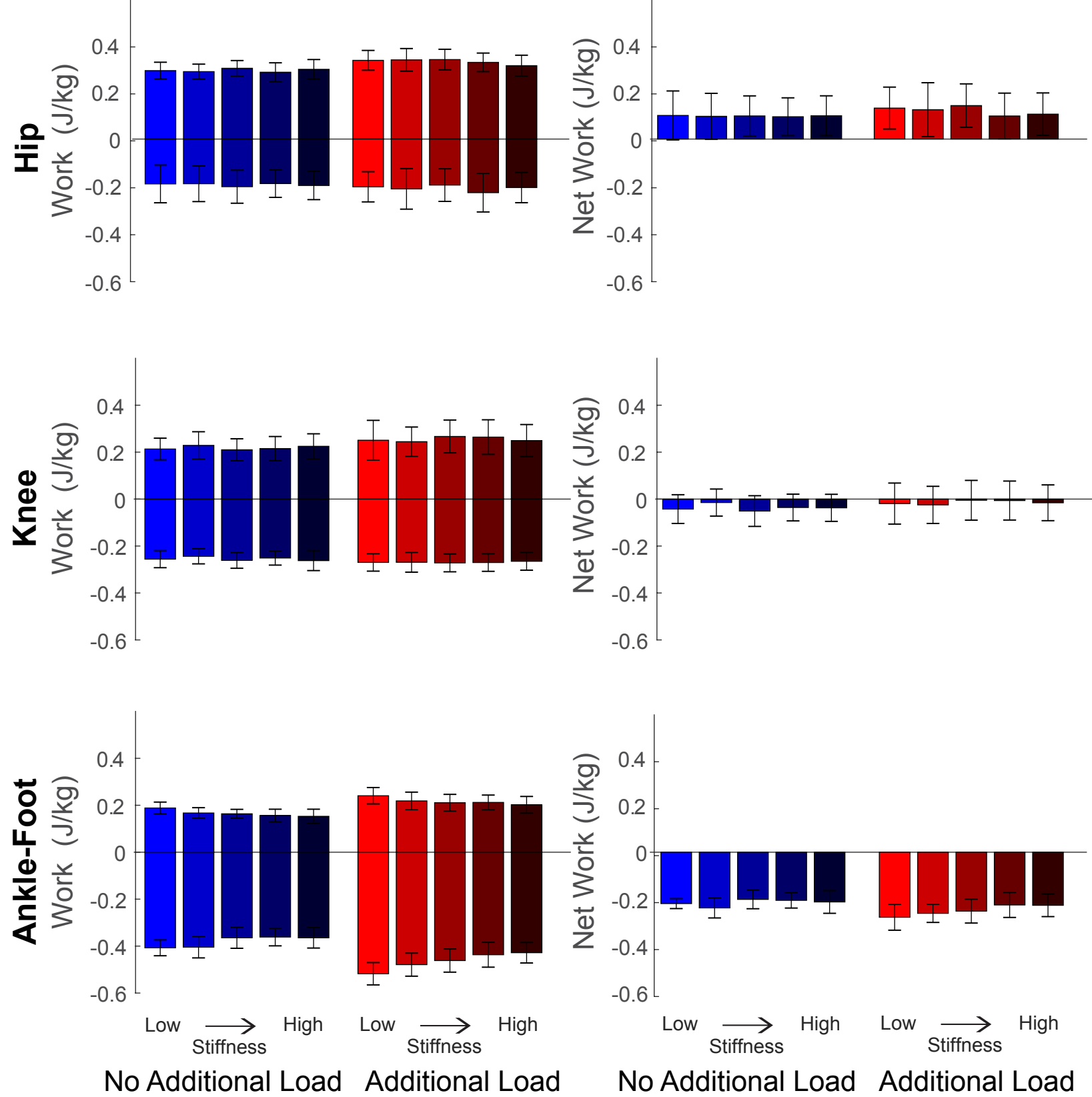

Supplement: Supplementary file 5 — Additional file 5: Figure S5. Prosthetic ankle-foot, and ipsilateral knee and hip work per stride for each condition. All values are normalized to biological body mass. Blue bars represent the no additional load, and red bars represent the additional load conditions. As the colors get darker, the stiffness values are increasing. [file 12984_2019_621_MOESM5_ESM.pdf]

# Hip

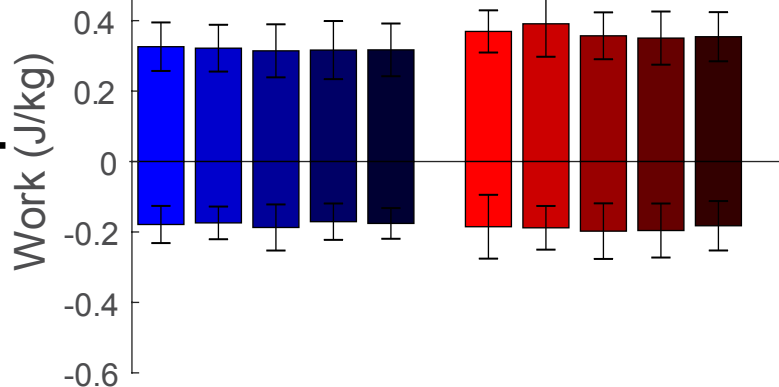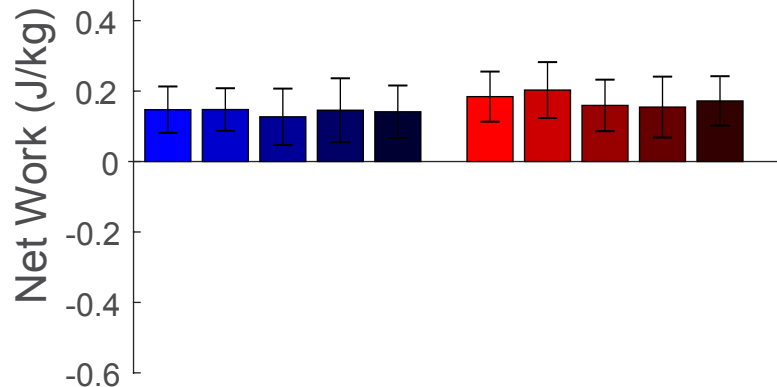

# Knee

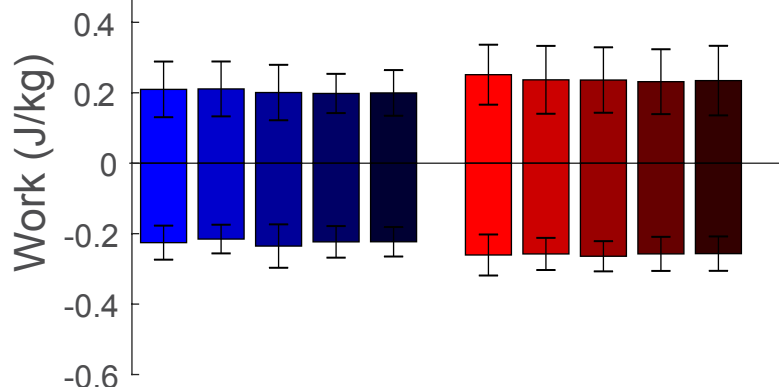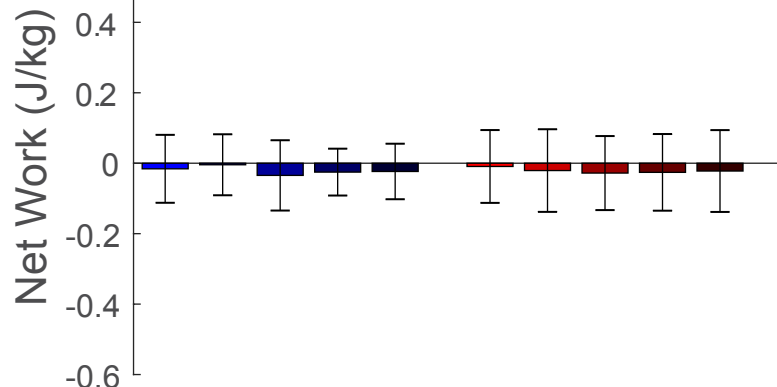

# Ankle-Foot

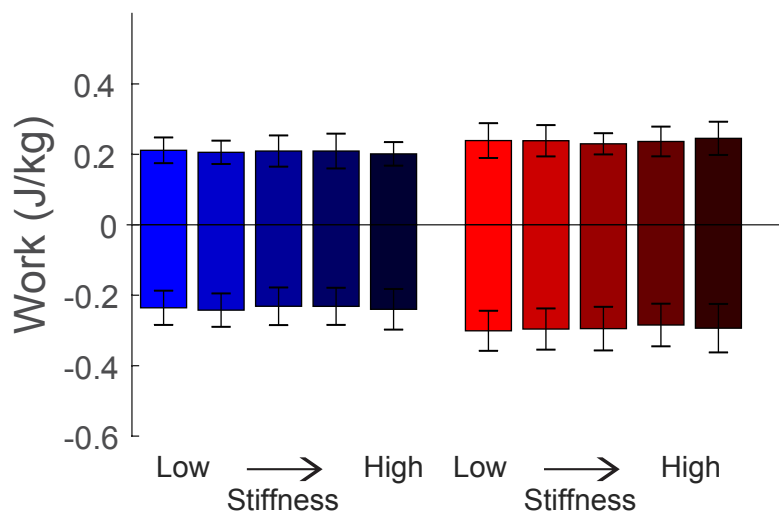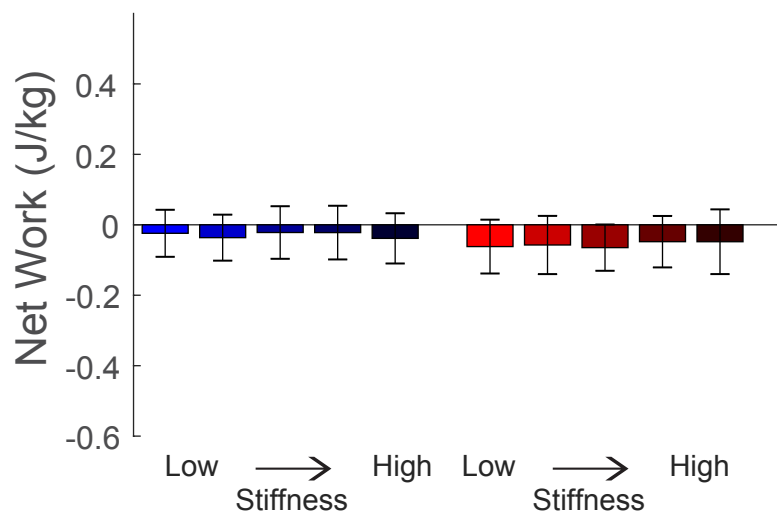

No Additional Load

Additional Load

No Additional Load

Additional Load

Supplement: Supplementary file 6 — Additional file 6: Figure S6. Contralateral ankle-foot, knee, and hip work per stride for each condition. All values are normalized to biological body mass. Blue bars represent the no additional load, and red bars represent the additional load conditions. As the colors get darker, the stiffness values are increasing. [file 12984_2019_621_MOESM6_ESM.pdf]

# Hip

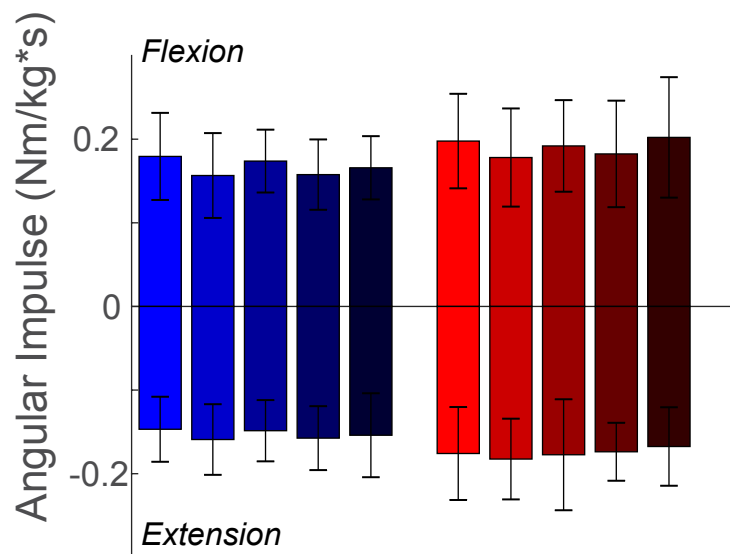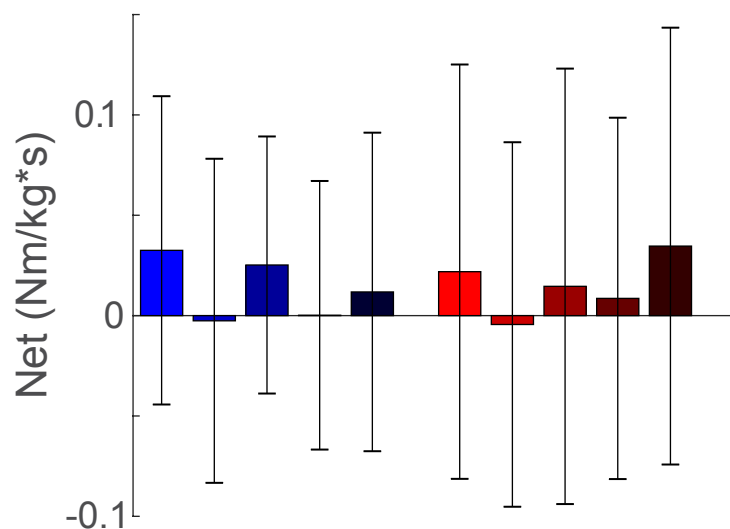

# Knee

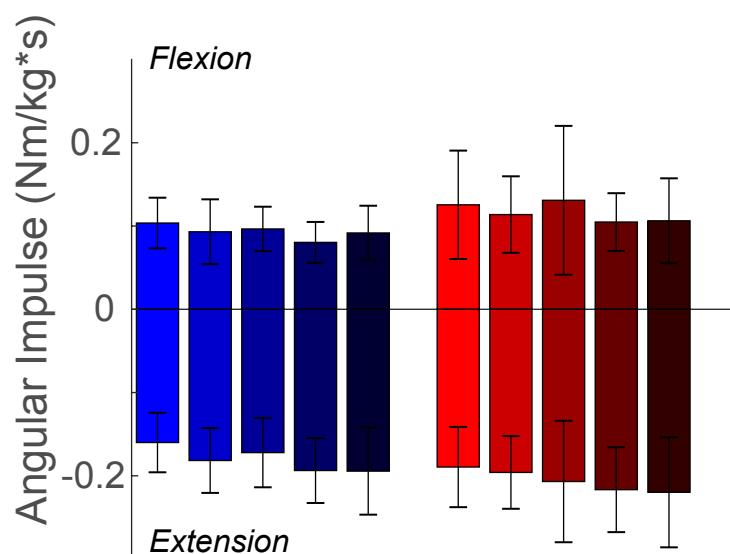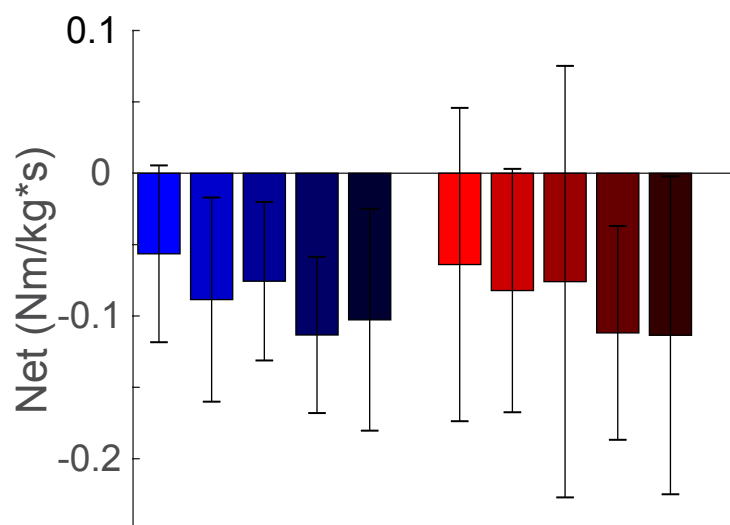

# Ankle

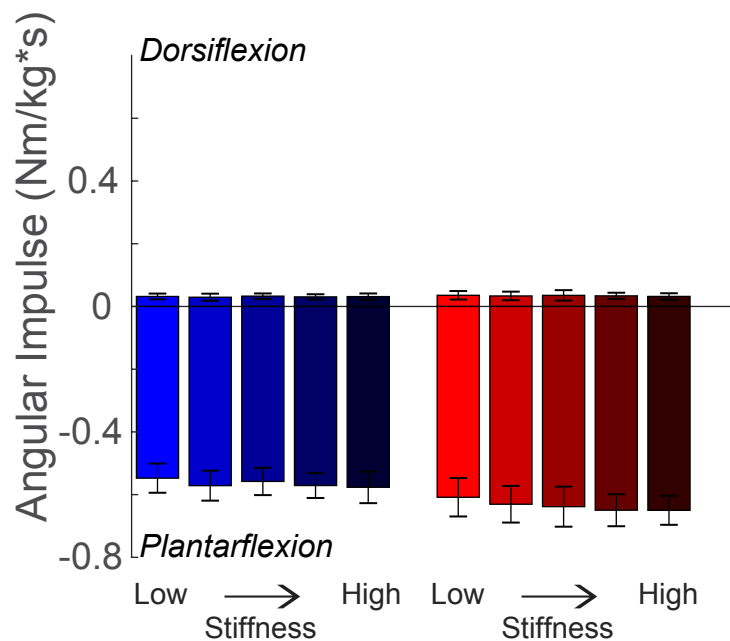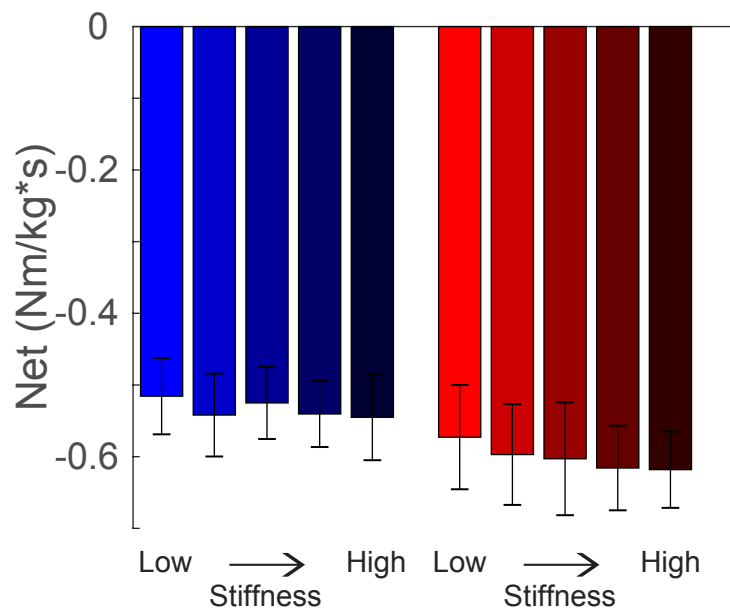

Supplement: Supplementary file 7 — Additional file 7 Figure S7. Ipsilateral ankle, knee, and hip angular impulse for each condition. All values are normalized to biological body mass. Blue bars represent the no additional load, and red bars represent the additional load conditions. As the colors get darker, the stiffness values are increasing. [file 12984_2019_621_MOESM7_ESM.pdf]

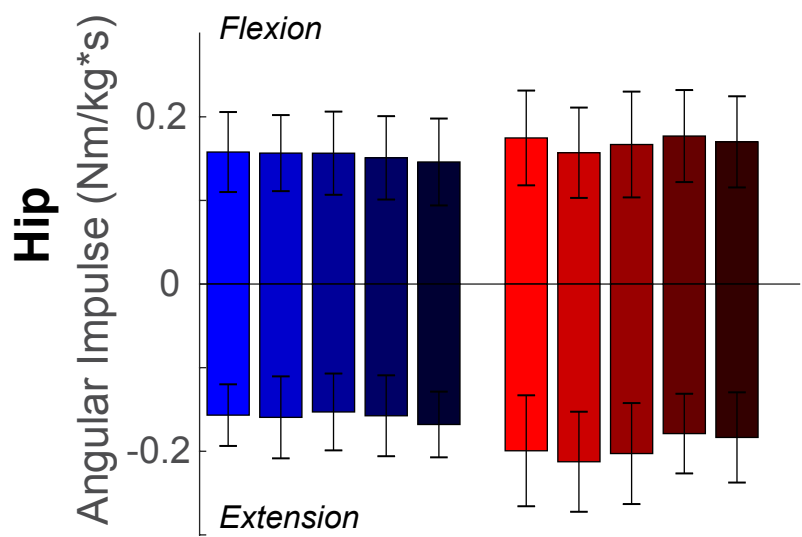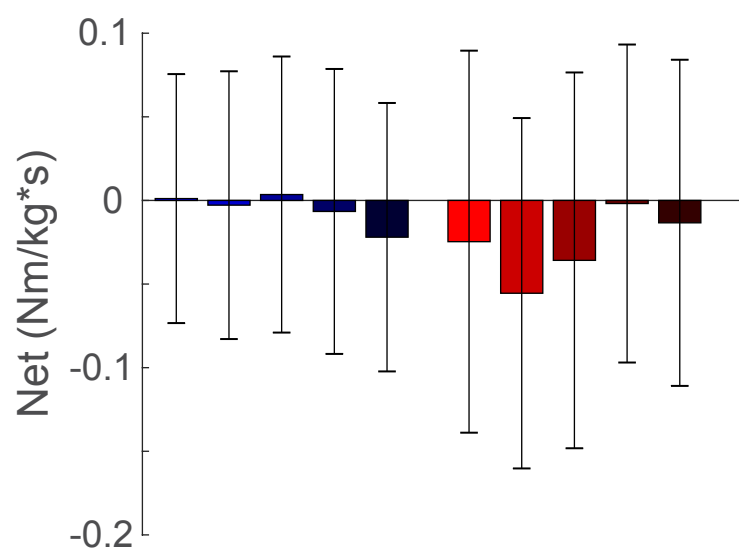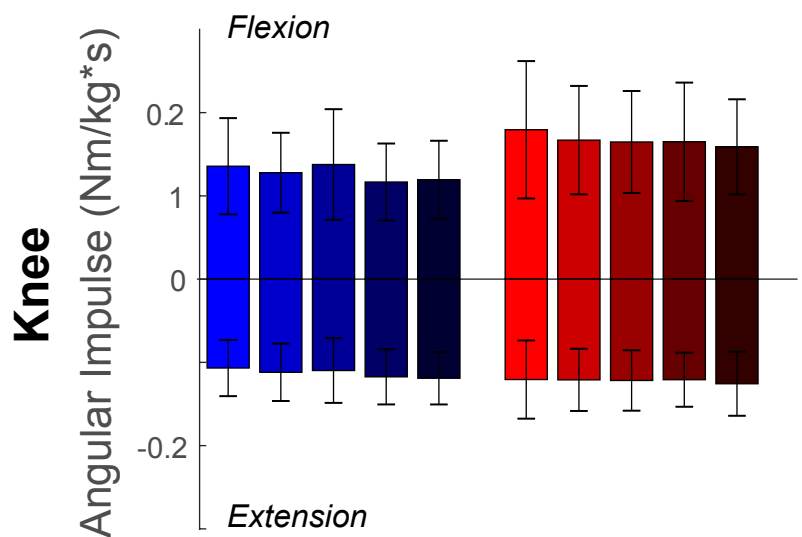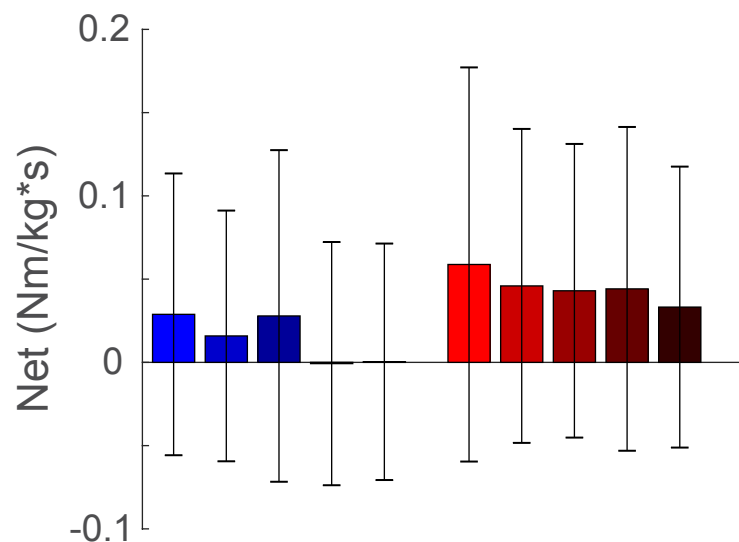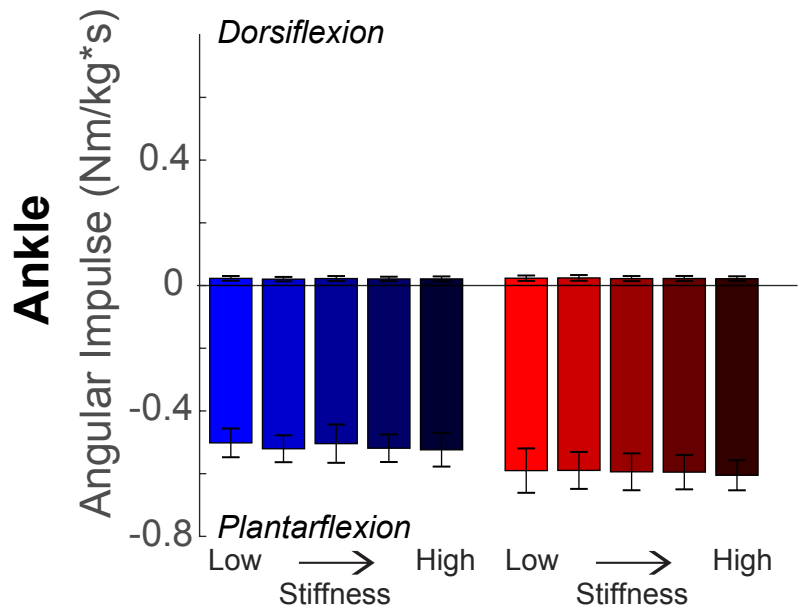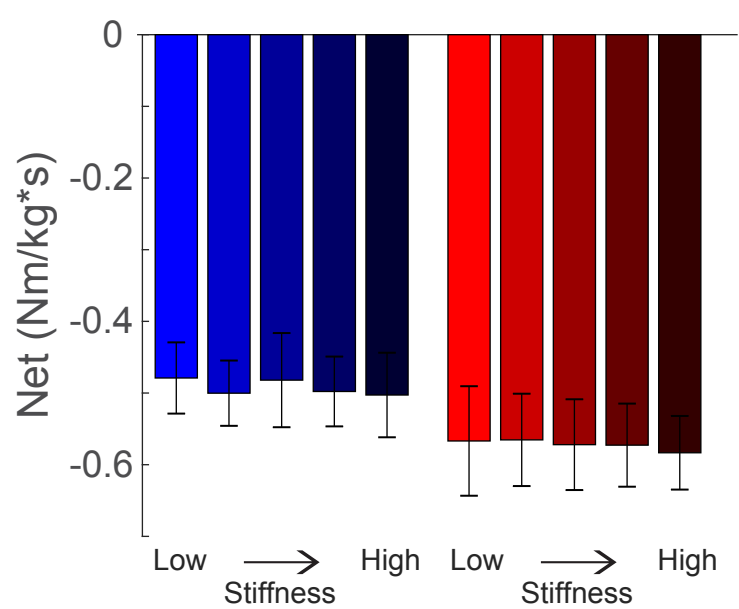

Supplement: Supplementary file 8 — Additional file 8: Figure S8. Contralateral ankle, knee, and hip angular impulse for each condition. All values are normalized to biological body mass. Blue bars represent the no additional load, and red bars represent the additional load conditions. As the colors get darker, the stiffness values are increasing. [file 12984_2019_621_MOESM8_ESM.pdf]

↕  
Less Difficult  
↕  
More Difficult

Perception = -1.91 - 0.23 • kl

- Additional load
- No additional load

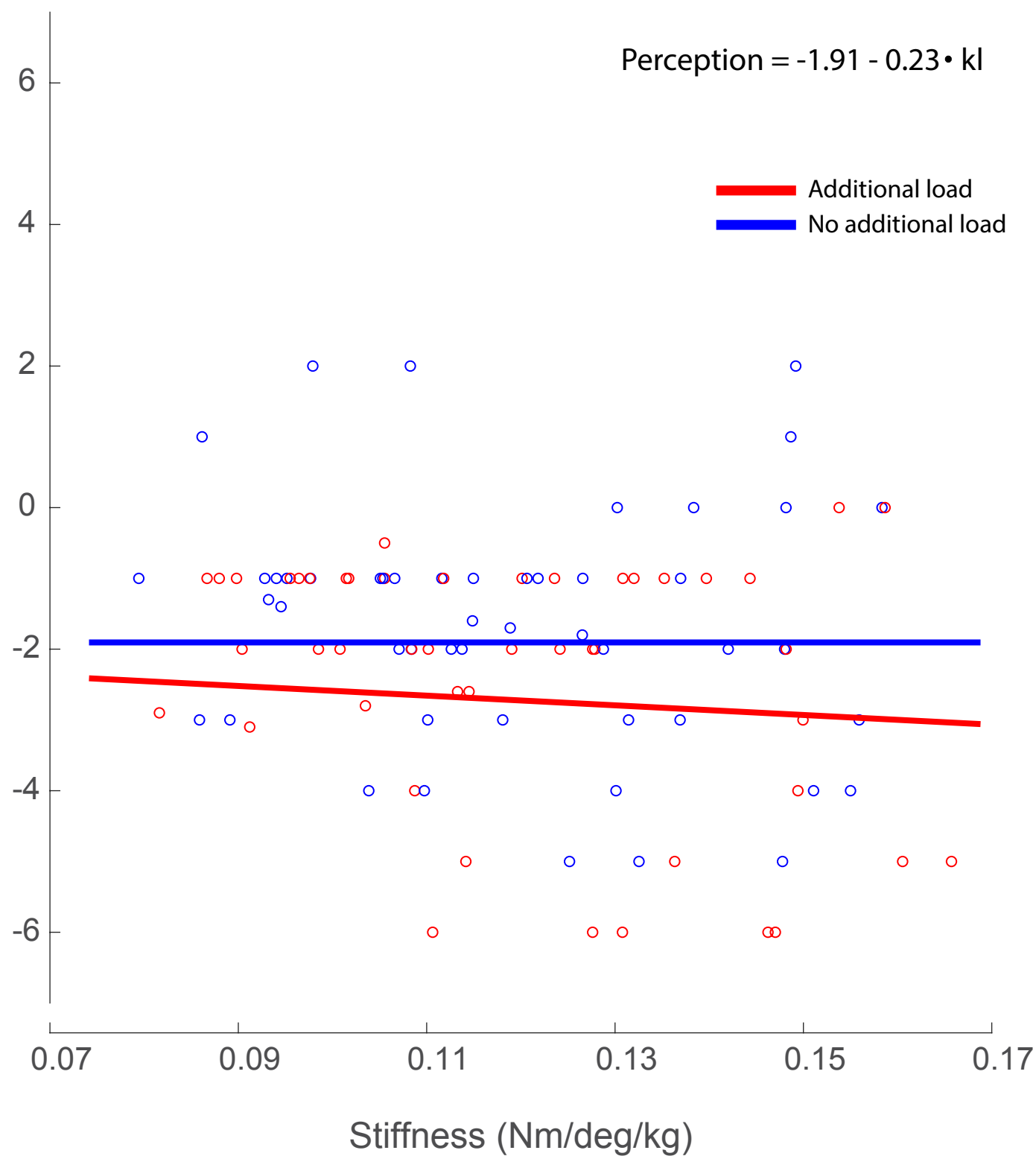

Supplement: Supplementary file 9 — Additional file 9: Figure S9. Participants’ perception of each conditions’ difficulty, compared to the actual stiffness of the condition. Each dot represents each participant’s individual data, and the solid lines are the predicted equation. The interaction of stiffness and load were significant predictors of the participant’s perception. Perception = − 1.91 − 0.23 ∙ kl Therefore, for the no load condition, participant’s did not perceive any difference in difficulty, but for the additional load conditions, the conditions seemed more difficult as stiffness increased. [file 12984_2019_621_MOESM9_ESM.pdf]

## No Additional Load

## Additional Load

Low  
Stiffness

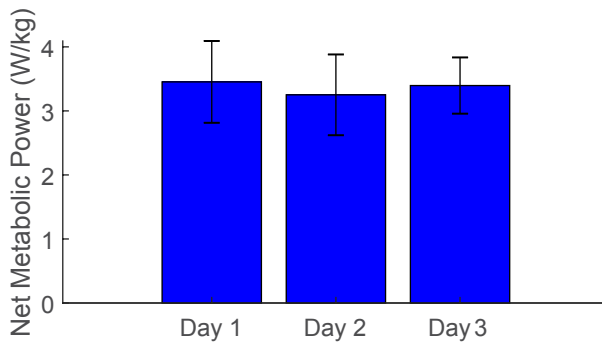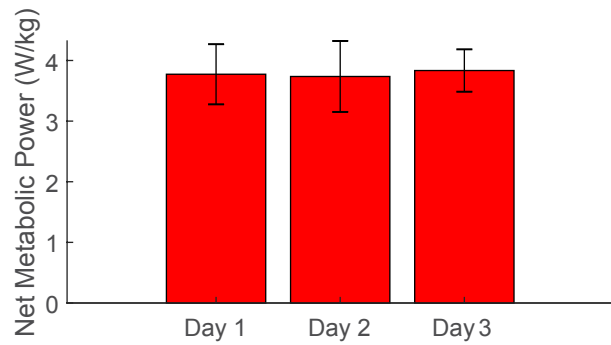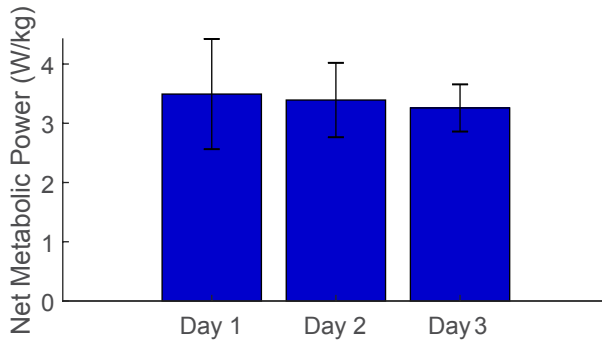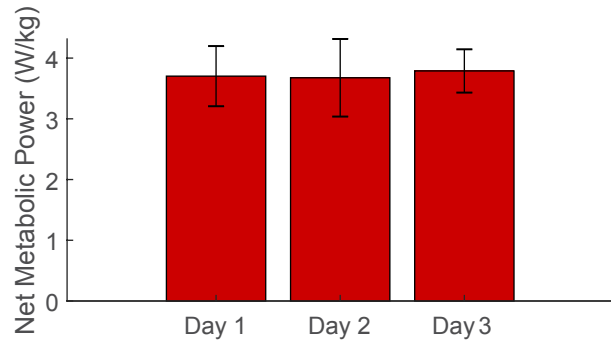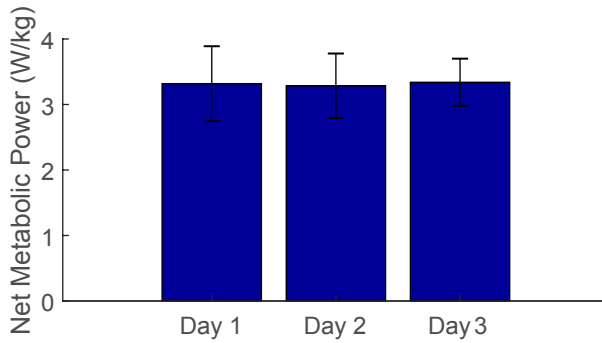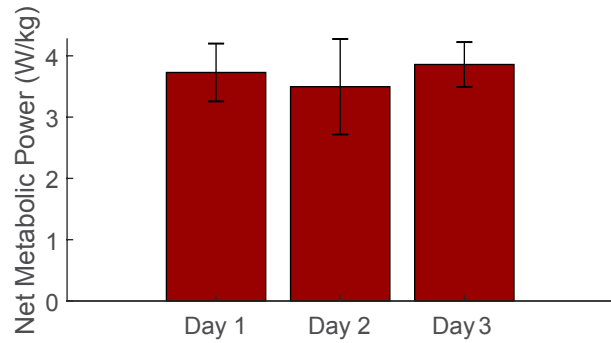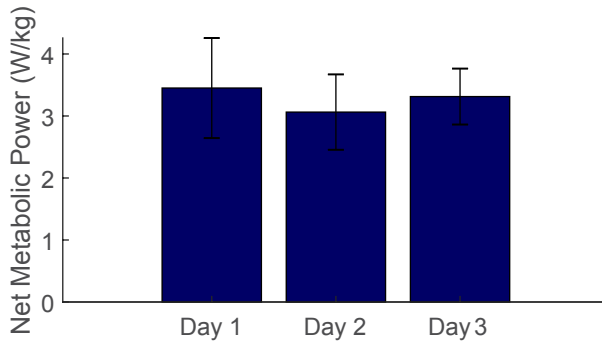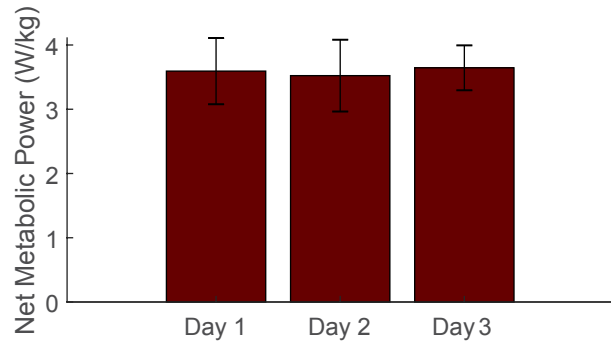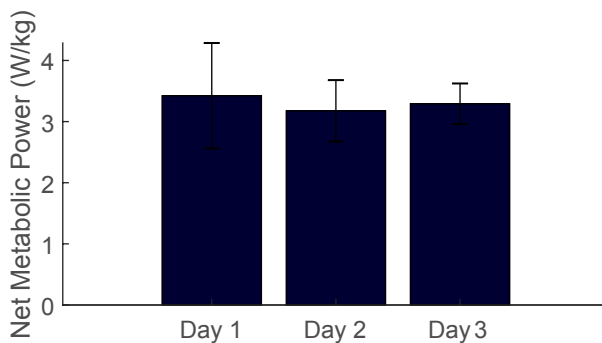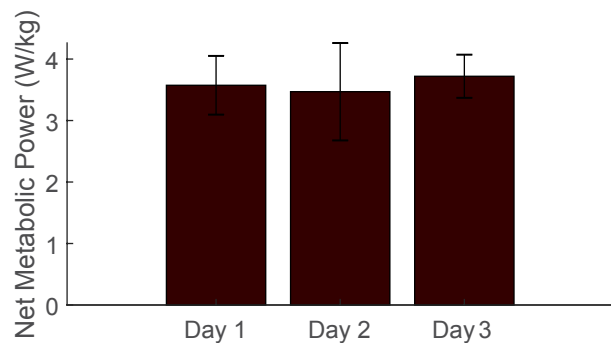

High  
Stiffness

Supplement: Supplementary file 10 — Additional file 10: Figure S10. Metabolic Cost for each condition across all 3 days. A paired t-test between the second and third day metabolic cost shows that there was no differences between the metabolic cost values for these days (p < 0.05). [file 12984_2019_621_MOESM10_ESM.pdf]
